# Quantifying and mitigating motor phenotypes induced by antisense oligonucleotides in the central nervous system

**DOI:** 10.1016/j.ymthe.2024.10.024

**Published:** 2024-10-28

**Authors:** Michael P. Moazami, Julia M. Rembetsy-Brown, Samantha L. Sarli, Holly R. McEachern, Feng Wang, Masahiro Ohara, Atish Wagh, Karen Kelly, Pranathi Meda Krishnamurthy, Alexandra Weiss, Miklos Marosfoi, Robert M. King, Mona Motwani, Heather Gray-Edwards, Katherine A. Fitzgerald, Robert H. Brown, Jonathan K. Watts

**Affiliations:** 1 RNA Therapeutics Institute, UMass Chan Medical School, Worcester, MA 01605 USA; 2 Department of Neurology, UMass Chan Medical School, Worcester, MA 01605 USA; 3 Department of Radiology, UMass Chan Medical School, Worcester, MA 01605 USA; 4 Department of Biomedical Engineering, Worcester Polytechnic Institute, Worcester, MA 01609, USA; 5 Program in Innate Immunity, Division of Infectious Diseases and Immunology, Department of Medicine, UMass Chan Medical School, Worcester, MA 01605 USA; 6 Department of Biochemistry and Molecular Biotechnology, UMass Chan Medical School, Worcester, MA 01605, USA

**Keywords:** oligonucleotides, gapmer ASOs, antisense therapeutics, phosphorothioate, neurotoxicity, toxicology, CNS delivery, neurotherapeutics, phosphate buffer, artificial CSF

## Abstract

Antisense oligonucleotides (ASOs) are emerging as a promising class of therapeutics for neurological diseases. When injected directly into cerebrospinal fluid, ASOs distribute broadly across brain regions and exert long-lasting therapeutic effects. However, many phosphorothioate (PS)-modified gapmer ASOs show transient motor phenotypes when injected into the cerebrospinal fluid, ranging from reduced motor activity to ataxia or acute seizure-like phenotypes. Using a behavioral scoring assay customized to reflect the timing and nature of these effects, we show that both sugar and phosphate modifications influence acute motor phenotypes. Among sugar analogs, DNA induces the strongest motor phenotypes while 2′-substituted RNA modifications improve the tolerability of PS ASOs. Reducing the PS content of gapmer ASOs, which contain a stretch of PS-DNA, improves their toxicity profile, but in some cases also reduces efficacy or duration of effect. We show that this acute toxicity is not mediated by major nucleic acid sensing immune pathways. Formulating ASOs with divalent ions before injection and avoiding phosphate-based buffers modestly improved tolerability through mechanisms at least partially distinct from reduced PS content. Overall, our work identifies and quantifies an understudied aspect of oligonucleotide toxicology in the CNS, explores its mechanism, and presents platform-level medicinal chemistry and formulation approaches that improve tolerability of this class of compounds.

## Graphical abstract

**Figure:**
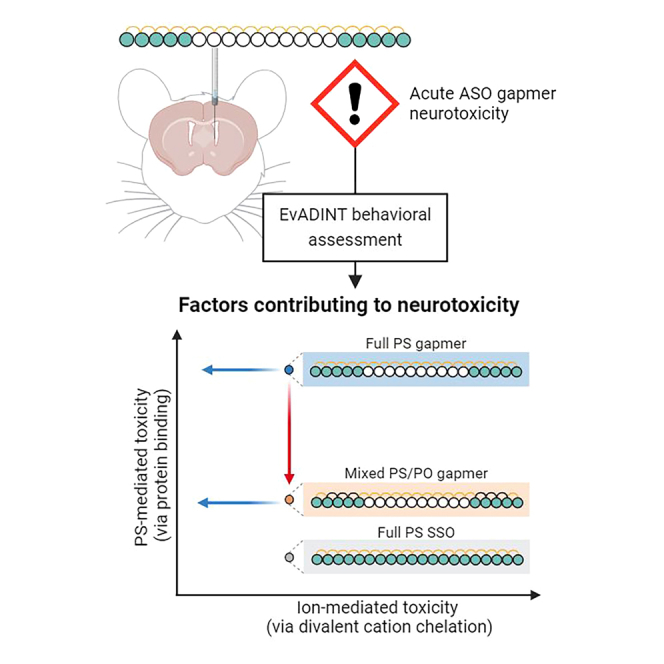

## Teaser

Watts and colleagues present a behavioral scoring assay to measure transient motor phenotypes induced by ASOs in the central nervous system. Reducing phosphorothioate content in ASO gapmers improves their tolerability in the CNS. Buffer formulation also affects acute phenotypes, with phosphate-based diluents showing lower safety than HEPES or lactate-based formulations.

## Introduction

Antisense oligonucleotides (ASOs) are emerging as an important therapeutic strategy for treating neurological diseases.^1^^,^^2^^,^^3^^,^^4^^,^^5^^,^^6^^,^^7^^,^^8^ However, we^4^^,^^9^ and others^10^^,^^11^^,^^12^ have reported adverse but transient motor phenotypes associated with ASO administration to the central nervous system (CNS) of mice. These behavioral phenotypes are typically most severe within the first 1–3 h after intracerebroventricular (i.c.v.) administration of the ASOs to mice. These phenotypes can vary in severity and may include seizure-like phenotypes, ataxia (and other gait disturbances), inability to maintain sternal posture, absence of a response to tactile stimuli, hyperactivity, and in extreme cases death. To date, there have been only a handful of studies studying these neuromotor phenotypes. Further assessment of these phenotypes is essential for understanding their mechanisms and developing more effective strategies to improve ASO safety.

Most of the behavioral scoring assays in the literature were not well aligned with rapid kinetics, reversibility and diverse phenotypes comprising both hyper- and hypoactivity. To objectively evaluate these transient phenotypes, we developed a quantitative scoring system called Evaluation of Acute Drug-Induced NeuroToxicity (EvADINT). Our EvADINT behavioral assay assigns weighted values to motor phenotypes observed directly following ASO delivery to the CNS. EvADINT scoring occurs at several time points over the first 24 h after compound administration, making it possible to sufficiently capture a range of ASO-induced phenotypes. This approach allows blinded experimenters to assess and compare the neuromotor phenotypes of diverse ASOs *in vivo*.

We apply the EvADINT scoring assay to probe the generality of this neurotoxic motor phenotype phenomenon, explore its mechanisms, and understand how it is affected by chemical modification and formulation strategies. We show quantitatively that mixed phosphorothioate/phosphodiester (PS/PO) backbone ASOs are less neurotoxic compared with full PS ASOs of the same sequence. Interestingly, the ASO-induced motor phenotypes of full PS ASOs are profoundly affected by the sugar modifications used: PS-modified DNA ASOs were the most toxic, while PS-modified ASOs composed entirely of 2′-O-substituted RNA (2′-O-methoxyethyl [MOE] or 2′-O-methyl RNA) were less toxic. We also show that the toxicity profile of mixed-backbone (PS/PO) ASOs can be improved by formulating the ASOs with buffered solutions containing divalent cations before injection into the CNS, and that phosphate-containing buffers induce higher phenotypes than buffers based on HEPES or lactate. In particular, researchers should avoid using phosphate buffers containing calcium ions, as these can form microscopic precipitates even when appearing as clear solutions to the naked eye.

We also present experimental results that shed light on the mechanism underlying the observed motor phenotypes. The fact that reducing PS content and divalent cation formulation improves safety suggests that neurotoxicity is due to a combination of PS-mediated protein binding and ASO-induced local divalent ion imbalances in cerebrospinal fluid (CSF). In addition, we demonstrate that neurotoxic ASO-induced motor phenotypes are not mediated by the major nucleic acid sensing innate immune pathways. Neuromotor phenotypes were observed in both small (mouse) and large (sheep) brains and PS reduction was helpful in both cases.

Progress in chemical modification of oligonucleotides has been profoundly important in enabling clinical success.^13^ Further improvements in modification and formulation of ASOs, as well as increased mechanistic understanding of the factors defining efficacy and toxicity, are essential to expand the therapeutic use of gapmer ASOs in the CNS.

## Results

### EvADINT scoring assay for acute behavioral toxicity

After administration of full PS ASOs into the CNS, we observed dose-dependent acute behavioral toxicity that varied from lethargy, lack of responsiveness and ataxia to hyperactivity, seizures, and, in extreme cases, death. This behavioral neurotoxicity was most striking in the first 1–3 h after administration; in some severe cases phenotypes were readily observed within minutes after administration. Even severely affected mice, unless they died, recovered fully by 24 h, and showed no further adverse effects. To describe our studies of acute toxicity in a robust way, we needed a way to quantify this acute toxicity.

Various protocols have been used to quantitate behavioral toxicity under the umbrella of a “functional observational battery,” with variations for both acute and longitudinal neurotoxicity.^14^ Regulatory documents such as the OECD guideline for neurotoxicity testing in rodents do not provide assay details that are well aligned with the transient toxicity seen after ASO administration to mice.^15^ Existing behavioral assays used by industry often lack transparency in their scoring methods. This hinders data comparison across research groups and ultimately progress in the field, thus underscoring the need for open assays with publicly accessible and validated scoring criteria.

We therefore developed a scoring assay optimized to quantify the transient motor phenotypes induced after intracerebral oligonucleotide injection. We call this assay EvADINT. In this assay, we monitored mice at multiple time points over 24 h after injection, assigning a score for various hyperactive and hypoactive behaviors as described in Table 1. Higher total overall EvADINT scores correspond to more severe and/or longer-lasting phenotypes.

**Table 1:** Breakdown of EvADINT scoring for quantification of acute motor phenotypes

Severity	None	Mild	Moderate	Severe	Death
Seizure^a^	0	10	15	20	75
Hyperactivity or other atypical motor behaviors^b^	0	5	10	15	

**Time required for (h):**	**0.5**	**1**	**2**	**4**	**24/no recovery**

Maintenance of sternal posture	0	4	8	12	20
Unstimulated movement	0	3	6	9	15
Movement without ataxia	0	2	4	6	10
Normal grooming/eating/nesting	0	1	2	3	5

Total score	–				

Scorers were blinded to groups during the observation period.

a Severity of seizures was ranked as follows: a severe seizure had a duration of >30 min and/or with constant or high intensity muscle contractions. A moderate seizure had a 10–30 min duration accompanied by moderate intensity muscle contractions and/or rapid and repetitive synchronous twitching with apparent short-term loss of consciousness. A mild seizure lasted <10 min and featured low intensity muscle contracts or short and infrequent bursts.

b Severity of hyperactivity or other atypical motor behavior was ranked as follows. Severe: >30 min duration with popcorning/jumping, constant. Moderate: 10–30 min duration, slight hopping, other atypical motor behavior. Mild: <10 min duration, uncoordinated movements, and twitching.

If a mouse died, it was given a maximum score of 75. Hyperactive phenotypes such as seizures, excessive movement, and other atypical motor behavior were scored depending on their severity. The greater the hyperactive phenotype severity, the greater contribution to the final EvADINT score. The remainder of the score was allotted to the severity and duration of hypoactive phenotypes (time elapsed before the animal was able to resume various aspects of normal mouse behavior). These observed hypoactive phenotypes were weighted according to their apparent severity. For example, maintenance of sternal posture was weighted more heavily than normal grooming since mice must be able to right themselves to carry out most other aspects of normal mouse behavior, and because maintenance of sternal posture is simpler than coordinated movement involved in eating, walking, or grooming. For similar intuitive reasons, seizures and death were weighted more heavily than the other factors such as latency to resume normal mouse behavior(s).

We varied the relative weightings of different factors in our EvADINT assay—death, seizures, and the various other behavioral observations—and observed that the toxicity rankings of different ASOs were maintained even when we assigned different weightings. Thus, while we chose these weightings based on an intuitive sense of relative severity and the need for a broad dynamic range, the EvADINT scoring assay is robust to variation in the precise weights assigned to each factor.

### Reduced PS content reduces acute neurotoxicity

Parallel work in our lab and by others has shown that mixed (PS/PO) backbone gapmers improved tolerability without loss of efficacy or duration of effect.^4^ The clinically approved antisense drug tofersen incorporates a mixed backbone design.^5^^,^^8^

**Figure 1:**
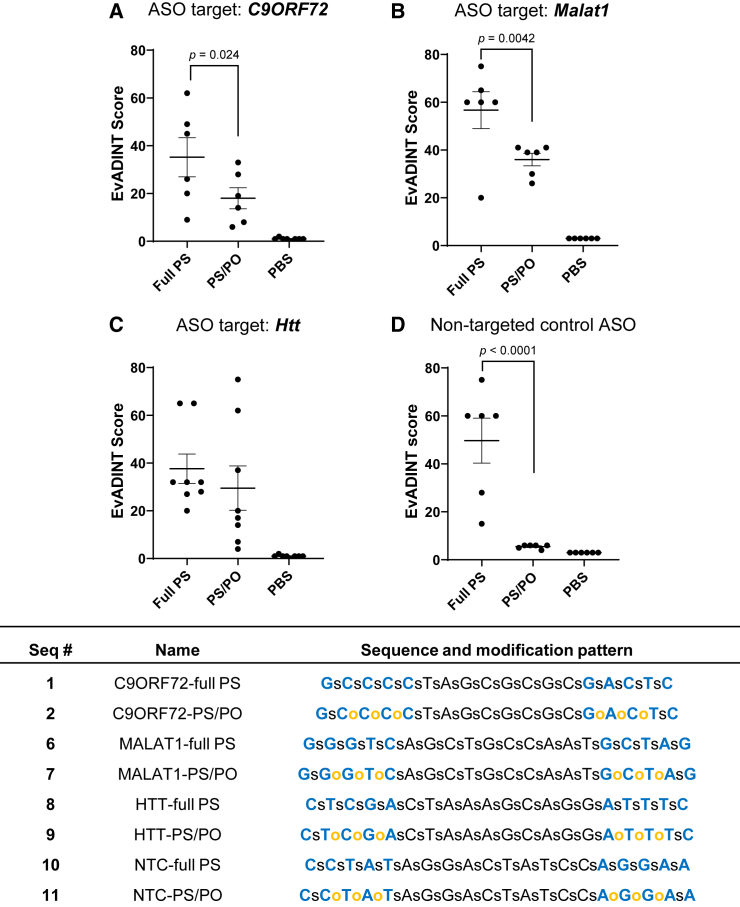
The tolerability of ASOs administered into the CNS is improved by modestly reducing the backbone PS content Mice were injected i.c.v. with 35 nmol of each ASO in 10 μL PBS (or with 10 μL PBS as control) and behavior was scored by a blinded investigator over the following 24 h using the EvADINT rubric. Sequences targeting (A) *C9ORF72*, (B) *Malat1*, or (C) *Htt*, or (D) a non-targeting control ASO, showed improvements in acute tolerability upon reducing the PS content. Each data point represents the EvADINT score from one mouse; *n* = 6–8; *p* values are calculated using one-way ANOVA. Full sequences and modification patterns associated with this figure are given in the corresponding table.

Our previously identified lead backbone design,^4^ and the mixed backbone design that we focus on exclusively in this study, contains a single PS linkage at each end of the ASO, followed by three PO linkages between the adjacent MOE-modified ribonucleotides. The principle underlying this design is that the combination of MOE sugars and PS linkages at the termini maintain robust exonuclease protection, while PS linkages are maintained throughout the remaining DNA (central) portion of the ASO to maintain endonuclease stability (if we included a PO linkage in the DNA region or at the bridge between the MOE and DNA regions, the efficacy dropped substantially).^4^ This design was also previously used for the *HTT*-targeted ASO tominersen.^16^ With the EvADINT scoring system established, we set out to quantify our observations of acute neurotoxicity and explore its generality and mechanism. We first validated that our EvADINT scoring system was able to distinguish the effects of reduced PS content under quantitative, blinded conditions. As expected, reducing the number of PS linkages led to increased tolerability in the CNS reflected in lower cumulative EvADINT scores comparing mixed PS/PO groups with full PS groups (Figure 1). This trend was independently reproduced across a total of four different ASO sequences providing evidence that our EvADINT scoring assay accurately captures the phenotypic effects of reducing the number of PS linkages.

### 2′-O-Alkyl sugar modifications reduce acute motor phenotypes

The combination of careful screening and the well-tolerated nature of fully 2′-modified ASOs in the CNS has allowed the rapid development and FDA approval of nusinersen^17^^,^^18^^,^^19^^,^^20^^,^^21^ as well as the first personalized ASO drug, milasen.^22^ Both nusinersen and milasen contain full PS backbones.^21^^,^^22^^,^^23^^,^^24^ These compounds are modified at each nucleotide with 2′-O-MOE; they are not gapmers and do not require a stretch of DNA because they function to redirect splicing rather than recruiting RNase H. We therefore wondered whether full PS-modified ASOs containing 2′-O-alkyl sugar modifications might show milder acute motor phenotypes than those containing DNA sugars.

**Figure 2:**
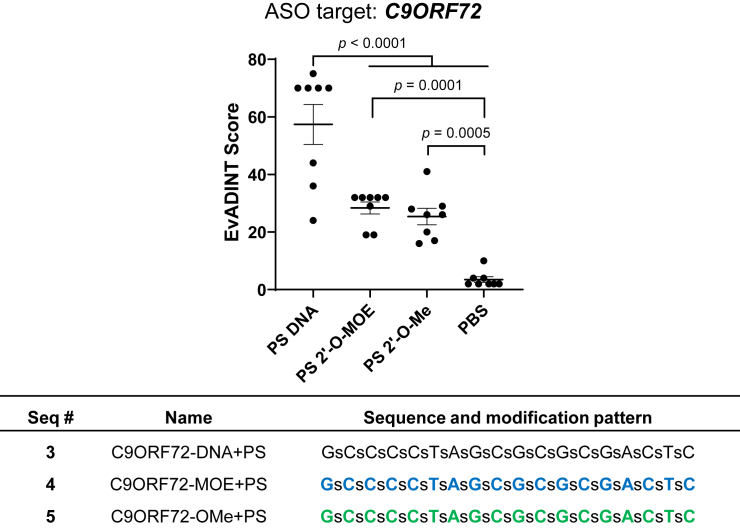
Fully PS ASOs containing 2′-modifications at each position are less toxic than those containing DNA at each position We injected 35 nmol of each ASO in PBS to the right lateral ventricle of mice, and recorded behavioral outcomes according to the EvADINT rubric. Each data point represents the EvADINT score from one mouse; *n* = 8; error bars represent SD; *p* values are calculated using one-way ANOVA. Full sequences and modification patterns associated with this figure are given in the corresponding table.

To study this question, we synthesized full PS ASOs modified *at every nucleotide* with DNA (PS-DNA), 2′-O-methyl RNA, or 2′-O-MOE, respectively (in contrast to the gapmer designs used in Figure 1). We suspended these in phosphate-buffered saline (PBS), injected them i.c.v. at 35 nmol/mouse and scored acute motor phenotypes using the EvADINT assay. The DNA-based oligonucleotide sequence **3** was dramatically more toxic than the two oligonucleotides containing 2′-modifications at every nucleotide (sequences **4** and **5**; Figure 2), while the two 2′-modified versions showed a dramatic reduction in motor phenotypes. We also carried out the comparison of full PS-2′-O-MOE with PS-DNA for a sequence targeting *Malat1* and saw the same dramatic difference in toxicity (data not shown: the *Malat1* experiment was carried out before we had established the quantitative EvADINT assay, but the clear difference we observed strongly suggests that the impact of sugar modification on toxicity is applicable to multiple sequences).

### PS ASO-induced acute neurotoxicity is not mediated by the major nucleic acid sensing innate immune pathways

The acute toxicity we observe is most intense in the first hour after injection of mice. This timing suggested to us that the acute motor phenotypes are not mediated by the innate immune system, since innate immune responses to nucleic acid stimuli typically do not peak until several hours after stimulation.^25^^,^^26^ Based on these differences in kinetics, we hypothesized that ASO-induced neurotoxicity was driven by interactions independent of immune activation.

**Figure 3:**
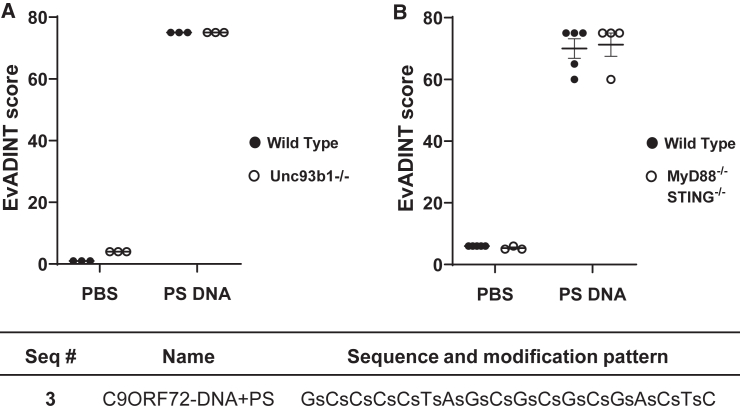
The acute neurotoxicity we observe is not mediated by Toll-like receptors 3, 7, or 9 or by the cGAS-STING pathway (A) Knockout Unc93b1^−/−^ mice (hollow dots) show an identical response to wild-type mice (filled dots), showing that the acute toxicity is not mediated by TLR3, TLR7, or TLR9 according to the EvADINT rubric. (B) Double knockout MyD88^−/−^ STING^−/−^ mice (hollow dots) show an identical response to wild-type mice (filled dots), showing that the acute toxicity is not mediated by TLR7, TLR9, or the CGAS-STING pathway. Each data point represents the EvADINT score from one mouse; *n* = 3–5; error bars represent SD. All PS-DNA samples were significantly different from all other samples in both panels (one-way ANOVA, *p* < 0.0001). The sequence and modification pattern associated with this figure is given in the corresponding table.

To confirm in a more direct way whether innate immune responses could play a role, we directly evaluated whether there was any contribution to this neurotoxicity from signaling through Toll-like receptors (TLRs) 3, 7, or 9, or the cGAS-STING pathway (Figure 3).

TLRs are a family of proteins that recognize pathogen-associated molecular patterns. TLRs 3, 7 (8 in human), and 9 specifically are endosomal sensors of nucleic acids and can induce a potent immune response *in vivo*. We delivered 35 nmol of this PS-DNA to *Unc93b1* knockout (KO) (*Unc93b1*^−/−^) mice.^27^ This strain lacks the *Unc93b1* gene, which encodes a chaperone protein required for intracellular trafficking of TLR 3, 7, and 9, three key nucleic acid sensing TLRs.^28^

Within minutes after anesthesia reversal, PS-DNA-treated *Unc93b1*^−/−^ and C57BL/6 WT mice exhibited severe seizures and did not survive to the end of the 24-h EvADINT observation period. These mice were assigned maximum EvADINT scores of 75 (Figure 3A), in line with the severe neurotoxicity previously observed with PS-DNA treatment (Figure 2). The fact that both *Unc93b1*^−/−^ and C57BL/6 wild-type (WT) mice exhibited excessive neurotoxicity after PS-DNA treatment provides strong evidence that neurotoxicity is not dependent on activation of TLRs 3, 7, or 9. *Unc93b1*^−/−^ and C57BL/6 WT mice treated with vehicle did not exhibit any adverse phenotypes. Vehicle-treated mice showed EvADINT scores with cumulative totals no greater than 4, indicating that neither the vehicle nor the surgical procedure drove the neurotoxicity.

We also evaluated the role of TLRs 7 and 9 more extensively as well as the cytosolic DNA sensing cGAS-STING pathway by testing ASO-induced phenotypes in mice lacking both MyD88 and STING (MyD88^−/−^ STING^−/−^ double KO mouse). This strain lacks signaling components for both endosomal and cytosolic nucleic acid sensing pathways: TLRs 7 and 9 signal through MyD88, whereas STING functions downstream of cGAS in cytosolic DNA sensing. Injection of a PS-DNA ASO into these double KO mice showed an identical response relative to a background-matched control mouse, confirming that the toxicity is not mediated by any of these nucleic acid sensors (Figure 3B).

Taken together, these experiments provide evidence that the acute toxicity, which is the focus of this work, is not mediated by the major nucleic acid sensing innate immune pathways. Of course, this finding does not preclude a role for other oligonucleotide-induced immune responses in the brain at longer time points, as described by other authors.^29^

### Pre-saturation with calcium reduces acute neurotoxicity

The composition of endogenous CSF is strictly controlled. Divalent cations such as calcium (Ca^2+^) and magnesium (Mg^2+^) are key components in CSF that support neural homeostasis. Phosphate linkages in ASOs can act as binding sites for CSF cations, and the polyanionic nature of ASOs leads to a high avidity for divalent cations in particular. The ability of ASOs to bind multiple divalent cations from CSF could theoretically therefore disturb delicate CSF ion balances. Furthermore, ASOs modified with PS are even more anionic in character at physiological pH than those with unmodified phosphates,^30^ which could further drive these ionic interactions.

Calcium is an essential mediator in neuronal signaling pathways^31^^,^^32^ and perturbations in Ca^2+^-dependent activities have been proposed as a mechanism for ASO-induced neurotoxicity.^10^^,^^11^^,^^12^ During i.c.v. injections, to keep volumes small, the ASO concentration is high (typically 1–4 mM). This is comparable with or higher than the concentration of Ca^2+^ in CSF, which is 1.3–1.4 mM^33^—and moreover, each polyanionic ASO could potentially chelate multiple Ca^2+^ ions. We wondered if reducing the PS content was reducing toxicity simply by reducing the tendency of the ASO to chelate Ca^2+^ ions.

At this point, our group and others routinely used PBS to solubilize ASO prior to CNS delivery. Standard PBS does not usually include divalent cations. Adding Ca^2+^ directly to PBS causes the irreversible formation of Ca^2+^-phosphate precipitates, although these can take several days or weeks to become visible to the naked eye following buffer preparation. Despite the temporal variability in the formation of visible precipitates, we wondered whether microscopic particles were forming earlier after buffer formation. Using dynamic light scattering, we confirmed the presence of precipitate particles (8–12 μm diameter) in sterile-filtered PBS prepared with 3.6 or 8 mM CaCl_2_ as early as 3 h after buffer preparation and sterile filtration (Table S1). No particles, however, were detected in PBS buffers containing only Mg^2+^ or in non-phosphate-buffered artificial cerebrospinal fluid (aCSF) solutions (discussed later) containing either Ca^2+^or Mg^2+^ prepared and analyzed under identical conditions (Table S1). These findings illustrate that, even at very early time points, and even when clear to the naked eye, Ca^2+^-containing PBS buffers comprise solid precipitate particles. Thus, addition of Ca^2+^ to PBS should not be used as a method for Ca^2+^ supplementation, since it is conceivable that injection of even micron-sized precipitates into the brain would run the risk of toxic phenotypes.

Calcium and phosphate are found together in some commercially available buffered solutions intended for clinical use in the CNS.^34^^,^^35^^,^^36^ One of the most well-known buffers is “Solution B″ from the work of the pioneering neurochemist K.A.C. Elliott, who initially developed the solution for replacement of CSF using surgery, and for whom it was a high priority to maintain physiological concentrations of phosphate, bicarbonate, and salts.^37^ Elliott described a method for buffer preparation including the preparation of three separate solutions and their combination in a specific order,^38^ and these solutions remain indefinitely free of visible (naked eye) precipitates.

As such, we wondered whether these solutions with their lower concentrations of calcium and phosphate (and potentially their careful preparation methods) would be free of microparticles. We purchased a vial of clinical-grade Elliotts B Solution and prepared aliquots of either the pure buffer or buffer containing ASO. We left some aliquots at room temperature, briefly heated others to 100°C, and subjected others to a round of freeze-thaw. The solution remained free of DLS-detectable particles when maintained at room temperature, but we observed the presence of micron-sized particles after brief heating to 100°C and in one of the replicates that had undergone freeze-thaw (see Table S2 and associated text). All of the Elliotts B Solutions remained clear to the naked eye.

To be able to test the role of calcium without concerns about calcium-phosphate microprecipitates, we next developed a buffer exchange protocol using ultrafiltration.^9^ Under this protocol, ASOs were added to Amicon cartridges (3-kDa mass cutoff) and washed with a 20 mM Ca^2+^ solution, to provide a slightly excess amount of calcium needed for PS linkage saturation. Importantly, to remove any residual Ca^2+^, the ASO was put through two washes with *in vivo* grade water before going through a final wash and resuspension in PBS.

**Figure 4:**
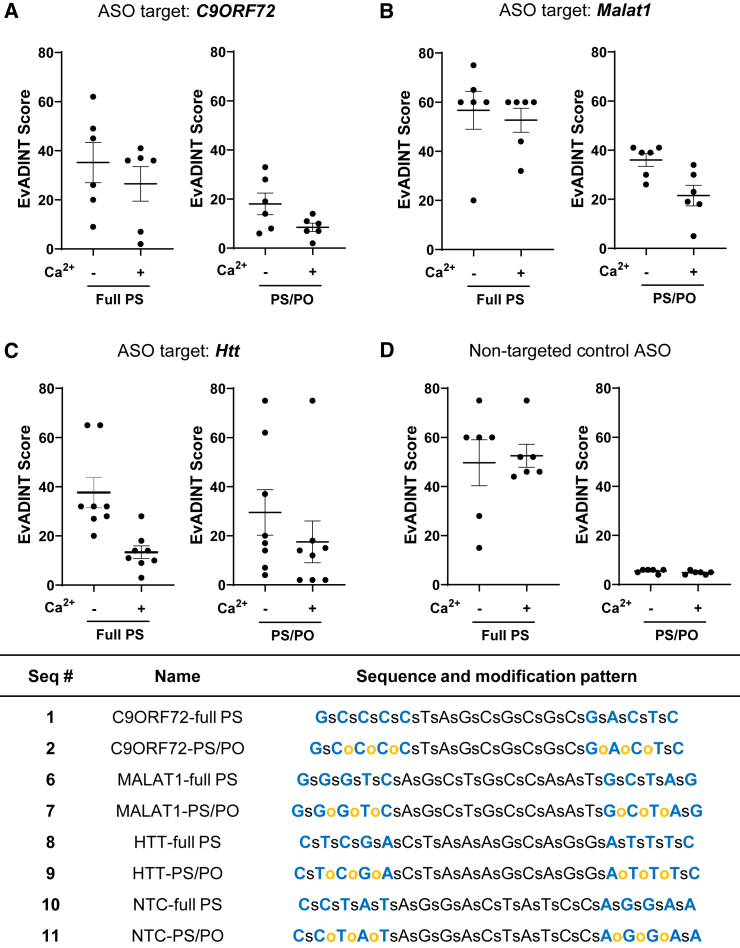
The tolerability of ASOs administered into the CNS is modestly improved using Ca^2+^ formulation Mice were injected i.c.v. with 35 nmol of each ASO in 10 μL PBS (or with 10 μL PBS as control) and behavior was scored by a blinded investigator over the following 24 h using the EvADINT rubric. Sequences targeting (A) *C9ORF72*, (B) *Malat1*, or (C) *Htt*, or (D) a non-targeting control ASO, showed improvements in acute tolerability upon reducing the PS content. Each data point represents the EvADINT score from one mouse; *n* = 6–8; error bars represent SD. *p* values are calculated using one-way ANOVA within GraphPad Prism software and represent per-comparison error rates. Full sequences and modification patterns associated with this figure are given in the corresponding table.

We chose a moderately high dose of 35 nmol ASO per mouse (equivalent to about 10 mg kg^−1^), somewhat higher than the dose typically required for effective gene silencing. Under these conditions, for our *C9ORF72*-targeted ASO, we observed that pre-saturation with Ca^2+^ led to modest improvement in acute tolerability in the CNS (Figure 4A). Interestingly, the improvement in tolerability occurred for both the full PS and the mixed backbone ASOs. Thus, the best tolerated ASO was the compound with reduced PS content, and which had also been pre-saturated with Ca^2+^.

These *C9ORF72* ASOs targeted the human transcript; the acute motor phenotypes were observed whether the ASOs had a target (as in the transgenic *C9ORF72* mouse models we used in the parallel work on therapeutic development for *C9ORF72*)^4^ or not (as in the WT mice used here).

To test whether these principles applied to other targeting and non-targeting sequences, we synthesized ASOs targeting the noncoding RNA *Malat*^38^ and the *Huntingtin* (*Htt*)^39^ mRNA as well as non-targeting control ASOs (sequences 6–9 and 10–11). For versions of these sequences with both full PS and mixed backbone formats, we compared simple formulation in PBS with formulation in PBS after Ca^2+^ pre-saturation. We injected these compounds i.c.v. into mice and observed motor phenotypes using the EvADINT assay. For the *Malat1*-targeting ASO (Figure 4B), we saw the same pattern as for the *C9ORF72*-targeted ASO—namely, there was a substantial improvement in tolerability upon reducing the PS content, and both the full PS and mixed backbone ASOs showed further improvement upon formulation with Ca^2+^.

For the *Htt*-targeted ASO, we saw broader variability in tolerability across the groups (Figure 4C). The improvement in tolerability of this mixed-backbone design was less clear for this sequence than for the other sequences. However, the improvement in tolerability from Ca^2+^ formulation was robust in the context of both backbone variants. Thus, there may be a sequence dependence to optimal backbone design, and it is clear that reducing the PS content in this way does not reduce the need to select good sequences.

Finally, we synthesized non-targeting control ASOs (sequences 10 and 11) and formulated them in the same way as the first three sequences. We injected these ASOs into mice and scored neurotoxicity using the EvADINT assay (Figure 4D). The patterns observed for this sequence are slightly different—in this case, Ca^2+^ formulation had little or no effect on toxicity and the reduction of PS content remained the most dramatic factor in toxicity.

Thus, pre-saturation of ASOs with Ca^2+^ showed a modest reduction in cumulative EvADINT scores across ASO sequences targeting *C9ORF72*, *Malat1*, and *Htt* and the design patterns we tested. This trend suggests Ca^2+^ chelation from endogenous CSF is partially contributing to the acute motor phenotypes (Figure 4).

However, this pre-saturation approach came with a significant drawback: a substantial amount (sometimes >50%) of the initial ASO amount was often lost during this buffer exchange, with the loss for some sequences being greater than others. ASOs have a tendency to aggregate in the presence of high concentrations of Ca^2+^. The amount of ASO in the flow-through measured between each wash step did not completely account for the total ASO lost, providing evidence that ASO was being trapped on the Amicon ultracentrifugation membrane. We adjusted the ASO concentration after calcium wash and before injection so that each mouse received the correct amount, but this variable loss of material significantly diminished the ease of applying our calcium wash protocol and made us consider formulation-based approaches.

### Formulating ASOs in a HEPES-based artificial CSF containing divalent cations

We next considered whether simply formulating ASOs in aCSF solutions already containing divalent cations would be a suitable alternative to Ca^2+^ supplementation by Amicon buffer exchange. Multiple artificial CSF formulations have been used to deliver drugs to the CNS.^34^^,^^37^ Generally, the ion composition of aCSF mimics that of endogenous CSF, however, an exact formulation is not well defined. These aCSF solutions typically include phosphate or bicarbonate as buffering agents.^37^^,^^40^ In the experiments shown in Figures 1, 2, 3, and 4 of this paper, we formulated the ASOs in PBS and encountered the limitations of phosphate in terms of calcium solubility. Bicarbonate buffers are in equilibrium with dissolved CO_2_ and thus are susceptible to pH changes, while the issues with phosphate have already been described above. Therefore, we considered alternative buffering agents.

We developed a novel aCSF formulation based on the zwitterionic buffering agent N-hydroxyethylpiperazine-N′-ethanesulfonic acid (HEPES). Using a non-phosphate-based solution allowed us greater versatility to incorporate different divalent cations while at the same time circumventing the Ca^2+^ phosphate precipitation issue highlighted in Table S1. As such, this buffer allowed us to test a range of concentrations of calcium and magnesium with all other variables kept constant.

In addition to this HEPES-based aCSF we also included lactate Ringer’s solution (LRS), a calcium-containing diluent readily available at USP grade. Lactate has only modest buffering capacity, being particularly vulnerable to pH increases after addition of base, but this is unproblematic as long as the ASO is at neutral pH before its dilution in LRS.

**Figure 5:**
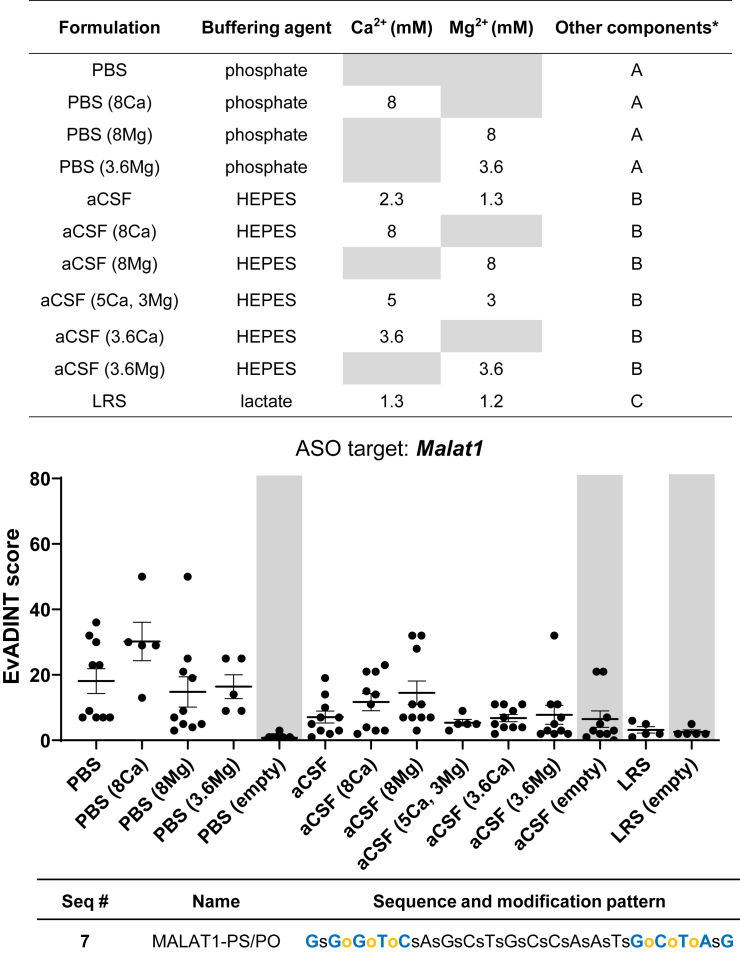
Impact of divalent ion and buffer formulation on neurotoxicity profile Table (on top) summarizing formulations and their components with abbreviations as follows: PBS, phosphate-buffered saline; aCSF, artificial cerebrospinal fluid; LRS, lactate Ringer’s solution; mM, millimolar. (∗) Denotes full list of components; (A) 155 mM NaCl, 2.9 mM Na_2_HPO_4_, 1.06 mM KH_2_PO_4_, (B) 137 mM NaCl, 5 mM KCl, 2.3 mM CaCl_2_, 1.3 mM MgCl_2_, 20 mM glucose, 8 mM HEPES, and (C) 100 mM NaCl, 3.7 mM KCl, 1.3 mM CaCl_2_, 1.2 mM MgSO₄, 28 mM NaHCO₃, and 20 mM NaC_3_H_5_O_3_. The tolerability of a mixed PS/PO ASO administered to the CNS in different formulations. Each data point represents the EvADINT score from one mouse; *n* = 5–10; error bars represent SD. Gray boxes highlight control dataset from empty vehicles. The sequence and modification pattern associated with this figure is given in the corresponding table (on bottom).

We tested a total of 11 different formulations that were modified from either standard PBS, our HEPES-based aCSF, or LRS (Figure 5). All 11 formulations were tested using the mixed PS/PO backbone *Malat1* gapmer (sequence 7), an ASO compound which provided us with a moderate baseline degree of neurotoxicity necessary to quantify any phenotypic differences based on changes in formulation. Mice were dosed with 25 nmol of PS/PO *Malat1* ASO using unilateral i.c.v. injections. Motor phenotypes were assessed under blinded conditions according to our EvADINT assay.

In comparing the EvADINT scores across these 11 different formulations, some important trends emerged (Figure 5). Generally, ASOs formulated in PBS were associated with higher EvADINT scores (greater neurotoxicity) when compared with HEPES-based aCSF formulations, indicating to our surprise that the choice of buffering agent—phosphate versus HEPES—may exacerbate or mitigate acute ASO neurotoxicity. ASOs formulated in LRS were associated with EvADINT scores comparable with HEPES-based aCSF.

ASOs formulated in 8 mM Ca^2+^ in PBS were associated with particularly high EvADINT scores (Figure 5). While not unexpected given the DLS results (Table S1), this outcome provided supporting evidence that, despite the absence of calcium-phosphate precipitates that are visible to the naked eye at the time of injection, the presence of microprecipitates may also cause adverse neurobehavioral effects. Due to the high toxicity of 8 mM Ca^2+^ in PBS, fewer mice were allocated to this condition. We had intended to test 3.6 mM Ca^2+^ in PBS in a subsequent study. However, based on the formation of micropreciptates also at this lower concentration (Table S1), we could not justify its administration *in vivo*. As expected, based on solubility properties, PBS with either 8 or 3.6 mM Mg^2+^ remained free of any visible or DLS-detectable microprecipitates; this coincided with a trend in lower EvADINT scores in treated groups. These results suggest that Mg^2+^, independent of Ca^2+^, may also play a role in mitigating ASO-induced neurotoxicity.

We noticed that mixed PS/PO ASO gapmers in either HEPES-based aCSF or LRS formulations had lower EvADINT scores on average compared with those in phosphate-based formulations. In mammals, there is approximately half the amount of phosphate in CSF compared with serum.^41^ In contrast to HEPES and lactate, phosphate ions are doubly charged at pH > 7.2 (i.e., the second pK_a_ of phosphate) and can thus have more complex interactions with divalent cations. Accordingly, Shahrokh et al. observed that phosphate buffers at pH > 7 caused adverse phenotypes in the context of protein delivery to the CNS.^42^ Thus, HEPES- or lactate-based solutions may be more favorable alternatives than phosphate for oligonucleotide delivery to the CNS, even beyond the problem of calcium-phosphate precipitation.

In summary, across all the tested formulations, the amount of total divalent cation concentration was not clearly associated with any advantages for reducing neurotoxic phenotypes mediated by mixed PS/PO ASO gapmers. Similarly, there were no obvious benefits conferred by using either Ca^2+^ or Mg^2+^ exclusively. Overall, the divalent ion concentration had a modest impact on reducing acute neurotoxic motor phenotypes. The three groups with the most consistently low scores were HEPES aCSF with 5 mM Ca^2+^ and 3 mM Mg^2+^, HEPES aCSF with 3.6 mM Ca^2+^ alone, and LRS used without further supplementation. Thus, while the presence of divalent cations may be advantageous, choosing a non-phosphate buffering agent also appears to have a beneficial effect.

### Impact of reducing PS content on efficacy

We previously observed for *C9ORF72*-targeted ASOs that this mixed backbone strategy maintained potency and efficacy as long as the PO linkages were between MOE nucleotides on both sides.^4^ To study the effect on efficacy of this backbone design across additional sequences, we compared the gene silencing of ASOs against *Htt* and *Malat1* in their full PS and mixed backbone versions. To be able to discern changes in compound efficacy, we chose a non-saturating (15 nmol) dose of each compound for this element of our study, and harvested brains after 3 weeks.

**Figure 6:**
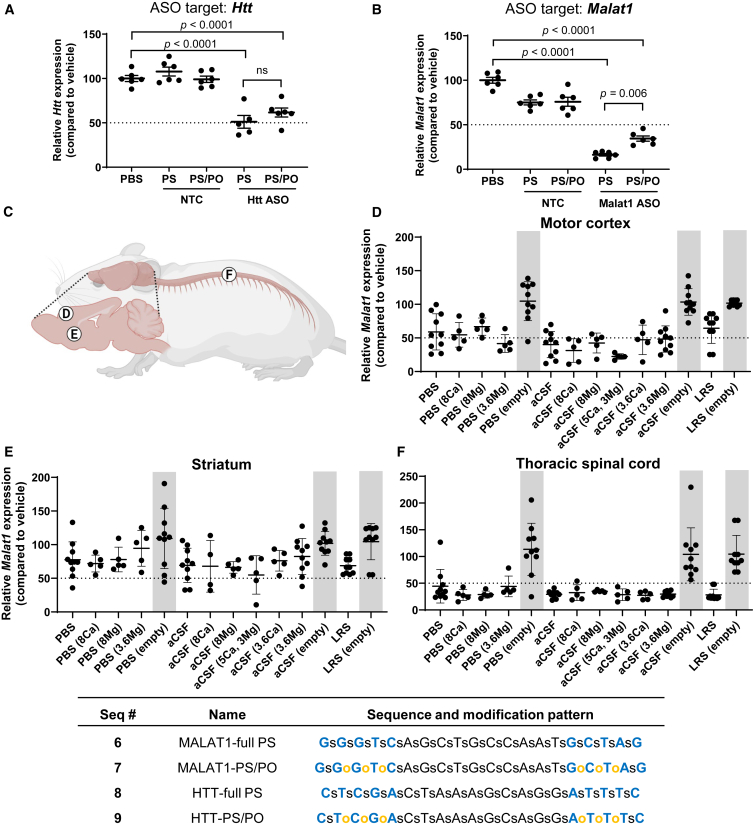
Effect of PS reduction and ion formulation on ASO efficacy Silencing of (A) *Htt* and (B) *Malat1* RNA 3 weeks after a 15 nmol dose of either full PS of mixed backbone (PS/PO) ASOs. (C) Schematic showing the approximate anatomical locations where samples were collected. Silencing of *Malat1* in (D) motor cortex, (E) striatum, or (F) thoracic spinal cord 3 weeks after a 25 nmol dose of mixed backbone (PS/PO) ASOs. Each data point represents the EvADINT score from one mouse; error bars represent SD; *p* values are calculated using one-way ANOVA. Gray boxes highlight control dataset from empty vehicles. Full sequences and modification patterns associated with this figure are given in the corresponding table. Panel (C) was created with BioRender.com.

We found that both the full PS and mixed backbone *Htt-* and *Malat1*-targeted ASOs significantly reduced their target mRNA expression, but, in contrast to our *C9ORF72* results,^4^ there was a trend toward reduced efficacy in the mixed backbone format relative to the full PS analogs (Figures 6A and 6B). It is not clear whether this results from a reduction in nuclease stability or a reduction in cellular uptake, since PS modification contributes to both factors. Nevertheless, this finding suggests that there is merit in exploring alternate backbone architectures, including next-generation mixed backbone designs,^43^^,^^44^^,^^45^ which might allow improvements in acute toxicity while maintaining or improving potency and efficacy.

### Impact of ion formulation on efficacy

Finally, we also wanted to evaluate whether different buffer or salt formulations had any impact on ASO silencing efficacy *in vivo*. Three weeks post-i.c.v. injection, we collected samples from motor cortex, striatum, and the thoracic region of the spinal cord and measured *Malat1* expression using quantitative PCR (qPCR) (Figure 6C). These CNS regions were selected because they are known to show differences in ASO distribution and silencing activity,^46^ allowing us to assess the full range of formulation-dependent effects.

Analysis of *Malat1* gene expression using qPCR revealed that the most prominent trends in efficacy were based on CNS region rather than formulation (Figures 6D–6F). Samples from thoracic spinal cord showed the greatest reductions in *Malat1* (Figure 6F) and were consistent across all formulations. Samples from the motor cortex (Figure 6D) showed some trends toward better silencing in the HEPES aCSF groups. The weakest reductions were observed in the striatum as expected,^46^ and comparable silencing was observed across all formulation groups (Figure 6E). Therefore, we can conclude that the efficacy of a gapmer ASO is not adversely affected by formulation in HEPES-based aCSF.

### The improved tolerability of mixed backbone ASOs applies to large brains

We wondered whether the acute toxicity we observed was an artifact of the small brain size of mice. This would make the concern significantly less relevant to researchers interested in therapeutic development of ASOs. To test whether the phenomenon applied to larger brains, we injected two sheep with full PS ASO (C9ORF72-full PS, sequence 1), and four sheep with the mixed backbone analog of the same sequence (C9ORF72-PS/PO, sequence 2), in LRS, at a dose of 2 mg kg^−1^.

Direct intrathecal injection, the route used for patients receiving ASO therapeutics, is not practical in sheep because of difficulty accessing the intrathecal compartment and because CSF tends to be expelled from the site where the dura is punctured, leading to poor uptake of ASO. Therefore, we used a technique whereby a microcatheter was threaded up through the intrathecal space and the ASO was delivered directly into the cisterna magna (see materials and methods). In all cases successful microcatheter navigation was performed into cisterna magna. Both intracisternal contrast injection and cone beam computed tomography confirmed the correct catheter position prior to ASO injection. Contrast material opacification was seen in the cisterna magna, around the cerebellum, and in the upper cervical spinal canal. No complication was observed in relation to catheter navigation or contrast injection.

None of the four sheep that were given the mixed backbone ASO (C9ORF72-PS/PO, sequence 2) showed evidence of abnormal motor phenotypes (0/4, 0%). In contrast, both sheep that were given full PS ASO (C9ORF72-full PS, sequence 1) showed hindlimb weakness and gait instability (wobbliness) within the first 24 h (2/2, 100%). Thus, the acute toxicity of full PS ASOs is not specific to mice but also applies to large brains. The ASOs were given in LRS, which confirms that the improvement in toxicity mediated by reducing PS content is at least partly distinct from the question of Ca^2+^ chelation, in larger brains (in this case, sheep) as in mice as described above.

## Discussion

The mechanism for the acute neurotoxicity of PS-modified ASOs is likely multifactorial. It is evident that reducing the PS content of ASOs can mitigate these neuromotor phenotypes. When we first posted our preprint on this work,^9^ an increasing number of papers were describing the use of gapmer ASOs in the CNS, many of which employed ASOs containing full PS backbones^39^^,^^47^^,^^48^^,^^49^^,^^50^ or those for which the modification pattern is not clearly disclosed.^3^^,^^51^^,^^52^^,^^53^ Some papers had begun to describe ASOs containing a mixture of PS and PO linkages for use in the CNS^6^^,^^16^^,^^50^^,^^54^^,^^55^^,^^56^^,^^57^^,^^58^^,^^59^; however, to the best of our knowledge, no comparative data on the neurotoxicology of these mixed-backbone ASOs relative to full PS ASOs had been presented.

While our work was being revised, three other groups also published work on oligonucleotide-induced motor phenotypes. First, in a study focused on LNA gapmer ASOs, Hagedorn et al. demonstrated that neurotoxicity could be at least partly replicated using an *in vitro* calcium oscillation assay in iPSC-derived neurons, and could be at least partly predicted based on sequence features, particularly guanosines at or near the 3′ end.^11^ Hagedorn et al. also confirmed in a conference presentation^60^ that the MOE gapmers described in our work showed the same toxicity ranking in his *in vitro* calcium oscillation assay as we observed in mice, suggesting the broad relevance of the calcium oscillation assay to identify neurotoxic sequences *in vitro*. This assay may thus increase the scalability of screening and reduce the number of animal studies needed.

Secondly, Yokota and co-workers sharpened a focus on intracellular calcium levels by showing that small molecules that promote calcium influx mitigate ASO-induced neurotoxicity, while those that hinder calcium influx worsen ASO-induced neurotoxicity.^10^ They identified a particularly important role for calcium channels and AMPA receptor, a neuronal ion channel and glutamate receptor, and they hypothesized that toxic ASOs may be binding to these cell-surface proteins.

Finally, Aronin and co-workers carried out a study of neuromotor phenotypes in the CNS with a focus on siRNAs.^12^ The duplex structure of siRNAs drives high avidity for divalent ions and, as such, they observed that divalent ion supplementation was highly effective at reducing siRNA-induced neuromotor phenotypes.

**Figure 7:**
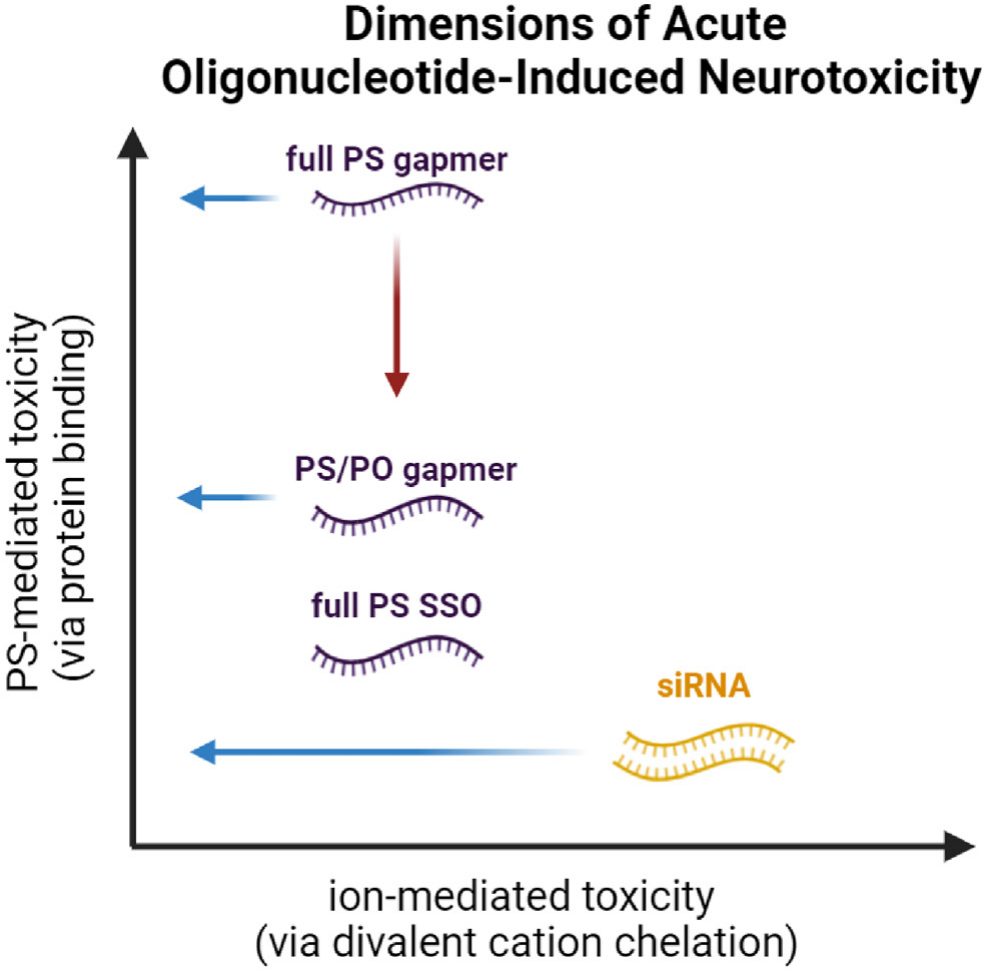
Proposed model for motor phenotypes associated with oligonucleotide-induced neurotoxicity Red arrow indicates mitigation of PS-mediated toxicity by the use of mixed (PS/PO) backbone ASOs. Blue arrows indicate mitigation of divalent ion-mediated toxicity by the use of divalent ion supplementation. siRNAs have been included to provide a more integrated model that broadly describes oligonucleotide-associated neurotoxicity, but these data have been described elsewhere.^12^ PS, phosphorothioate; PO, phosphodiester; SSO, splice switching oligonucleotide. Created with BioRender.com.

Putting together all of the data in our own work and these other studies, we propose a model (Figure 7) in which both protein binding (y axis) and ion binding (x axis) can contribute to neuromotor phenotypes. For ASOs, protein binding is likely to dominate, while the more rigid structure and high divalent ion binding of duplexes means that ion binding may dominate the toxicity of siRNAs.^12^ We discuss each of these two “axes” in more depth in the next two sections.

### PS content and sugar modification show the importance of protein binding in driving acute neurotoxicity

At least part of the acute neurotoxicity of PS ASOs is likely to be mediated by binding to cell-surface proteins. PS-DNA shows extensive binding to a variety of proteins,^61^^,^^62^^,^^63^^,^^64^ including cell-surface and trafficking proteins.^65^^,^^66^^,^^67^^,^^68^^,^^69^ PS reduction is also well known to reduce protein binding: e.g., after systemic administration, an earlier generation of mixed-backbone modification ASOs showed reduced binding to proteins such as complement pathway members and clotting factors.^70^^,^^71^^,^^72^^,^^73^

Our work also demonstrates that 2′-modified PS-RNA exhibits lower neurotoxicity compared with PS-DNA. These results align with previous research showing that PS-RNA reduces non-specific protein binding compared with PS-DNA.^74^ For example, one study found that PS-MOE bound various plasma proteins with 3- to 50-fold lower affinity relative to PS-DNA.^75^ In another study, PS-2′OMe gapmers of two different sequences showed about 2.5-fold lower-affinity protein binding than PS-DNA of the same sequence.^69^

The fact that PS-DNA shows higher acute toxicity than the corresponding 2′-modified nucleotides (Figure 2) suggests that a major area of focus for nucleic acid chemists should be the development of sugar or phosphate-modified DNA analogs that elicit robust RNase H cleavage while reducing the incidence of motor phenotypes when administered to the CNS.

### Divalent cation supplementation and buffer choice can also be beneficial for mitigating neurotoxicity

Ca^2+^ chelation has been responsible for unexpected toxicities in other classes of drugs.^76^ Indeed, other studies have documented perturbations in calcium dynamics, including changes in intracellular free Ca^2+^ and reduced Ca^2+^ oscillations, following ASO treatment.^10^^,^^11^ These observations suggest that dysregulation of calcium homeostasis is a potential mechanism underlying ASO-mediated acute neurotoxicity. In our work, calcium supplementation reduced toxicity less consistently than reductions in PS content.

To test the role of calcium while minimizing the possibility of undesirable calcium-phosphate microprecipitates, we developed a new aCSF solution that featured HEPES instead of phosphate as a buffer. To the best of our knowledge, a HEPES buffer has not been used in the CNS before. HEPES exhibits weak interactions with metal ions,^77^^,^^78^ which, together with the strong buffering capacity of HEPES, made it an ideal alternative buffer for our CNS work.

Interestingly, we found that, compared with PBS, ASOs formulated in HEPES-based aCSF exhibited trends of lower EvADINT scores compared with ASOs formulated in PBS that were more consistent than even the improvements associated with divalent ion supplementation. The lower EvADINT scores associated with HEPES prompted us to ask if this was due to an intrinsic benefit of HEPES or a specific drawback of phosphate.

To differentiate this, we included LRS as a control. Unlike HEPES, lactate is a natural cellular metabolite, but its buffering capacity is less robust under alkaline conditions. However, if the ASO’s pH is carefully adjusted before formulation, drastic changes in pH may be avoided. Ultimately, we found that ASOs in either HEPES-aCSF or LRS showed similar trends in EvADINT scoring and represent viable formulation options. Furthermore, this provides evidence that phosphate itself might be less ideal for ASO delivery, consistent with previous observations by Shahrokh et al. that phosphate buffers of pH > 7 were associated with adverse effects in the context of protein delivery to the CNS.^42^

### Acute neurotoxicity is not mediated by major nucleic acid sensing immune pathways

Our experiments document that ASO-induced transient motor phenotypes are not a downstream consequence of innate immune signaling through the major nucleic acid sensing immune pathways.

Endotoxins are potent immune stimulating lipopolysaccharides that are not readily eliminated through common laboratory sterilization techniques. Some groups have attributed neurotoxic phenotypes to endotoxin contamination, but our experiments using the KO mouse strains in Figure 3 argue against this since endotoxin recognition signals through MyD88. In addition, we directly measured endotoxin levels (Endosafe nexgen-PTS spectrophotometer, Charles River) on the reagents used in our ASO formulations and on many of the final ASO preparations. The samples tested were below the threshold limit for endotoxins in CNS applications, reinforcing that the neurotoxicity was also not driven by endotoxin-mediated immune activation.

### Oligonucleotide purification byproducts can also induce neurotoxicity

Besides the factors described above, without careful attention to detail, small-molecule contaminants can also be an unexpected source of acute neurotoxicity. In one experiment not included above, we observed higher than expected toxicity and, using a solution of methylene blue, found that the specific batch of ASOs used in that experiment contained residual sodium perchlorate, a common agent used in ion-exchange HPLC purification (see Figure S2 and associated text).^79^ We strongly suggest that groups carrying out anion-exchange chromatography for oligonucleotides to be used *in vivo* adopt sodium bromide or other system instead of perchlorate.

### Neurotoxicity and hepatotoxicity likely occur through distinct pathways

The acute motor phenotypes discussed in this work do not appear to be related to other types of ASO-induced toxicity—such as immune stimulation (as discussed above) or liver toxicity. In ongoing work in our group, we have come across sequences that are well tolerated in terms of acute motor phenotypes, but still exhibit liver toxicity, and vice versa. The timing of these effects is also very different: with acute motor phenotypes strongest in the first hour (perhaps driven by binding to cell-surface receptors as discussed above), innate immune stimulation peaking at 1–2 days (driven by binding to TLRs and cytosolic nucleic acid sensors), and liver toxicity evident from 1 day to several days after dosing (this liver toxicity can be driven by factors including off-target cleavage of partially matched targets^80^^,^^81^ and mislocalization of paraspeckle proteins to nucleoli^82^).

### Limitations of the study

We acknowledge that mice have drastically different CNS structures and CSF flow dynamics compared with humans. The average adult mouse has around 40 μL of CSF^83^; the average adult human can have up to 150 mL of CSF, almost 4,000 times the volume of total mouse CSF. Although overall CSF ion concentrations between mouse and human are similar, species differences in total volume and flow pathways necessitate markedly different ASO doses to achieve efficacy.

In our experiments, we used an injectable agent to immediately reverse the effects of our surgical anesthesia (see materials and methods). This better reflects a clinical context where patients are not under anesthesia when given ASOs intrathecally. We began behavioral observations after reversal which ensured that we could observe any neurotoxic phenotypes that occurred shortly after ASO delivery. Oligonucleotides administered under different anesthetics, such as avertin or the inhalant isoflurane, have shown different neurotoxicity profiles experimentally.^12^ Although we have not extensively explored anesthetic-dependent effects of neurotoxicity, we hypothesize there may be differences in neurotoxicity related to the rate of anesthesia metabolism.^84^^,^^85^ For example, the effects of anesthesia with isoflurane wear off more gradually; this may suppress some acute neurotoxic phenotypes.

The development of next-generation mixed-backbone ASOs is an active area of research in our group.^43^^,^^86^ By identifying factors that mediate acute neurotoxicity, we also define actionable ways to address them. As we continue to test these strategies, researchers can more readily implement our HEPES-based aCSF or LRS formulations in combination with reducing the number of PS linkages to improve the therapeutic index of ASO gapmers. This will help pave the way for safer and more effective ASO gapmers in the CNS and expand the therapeutic applications of ASOs as treatments for neurological diseases.

## Materials and methods

### Oligonucleotide synthesis

All oligonucleotides were synthesized using a Dr. Oligo 48 synthesizer using standard methods. Phosphoramidites were purchased from ChemGenes and diluted to 0.1 M in acetonitrile. Sulfurization was accomplished using DDTT (0.1 M, ChemGenes). Benzylthiotetrazole (0.25 M in acetonitrile, TEDIA) was used as activator. All cytosine residues were 5-methylcytosine.

ASOs were deprotected in concentrated aqueous ammonia (30% in water) at 55°C for 16 h and characterized by liquid chromatography–mass spectrometry (LC-MS). After HPLC purification, final desalting was effected by ultrafiltration/diafiltration (3× water wash) in a 3-kDa cutoff Amicon centrifugal filter (Millipore).

The sequences and modification patterns of oligonucleotides are shown in tables within their corresponding figures. For modifications: bold blue, MOE; bold green, 2′-OMe; black, DNA. Lower case letters s and o refer to PS and PO linkages, respectively. NTC, non-target control ASO.

### Animal experiments

All animal procedures were conducted according to the Institutional Animal Care and Use Committee (IACUC) protocols of the University of Massachusetts Chan Medical School.

### STING/MyD88 double KO mice

MyD88^−/−^ mice on C57BL/6 background^87^ were obtained from S. Akira (Osaka University, Osaka, Japan). STING^−/−^ mice on C57BL/6 background^88^ were originally from G. Barber (University of Miami, FL) and obtained from D. Stetson (University of Washington, Seattle, WA).The two strains were intercrossed to generate MyD88^−/−^ STING^−/−^ double KOs. The mice were bred and maintained under pathogen-free conditions in our animal facility. The MyD88 and STING deficiencies were confirmed by performing PCR on DNA obtained after digesting a tail snip. For MyD88, specific primer pairs were used to distinguish the WT or KO allele in two separate reactions. Reaction 1 with primer sequences AGC CTC TAC ACC CTT CTC TTC TCC ACA and AGA CAG GCT GAG TGC AAA CTT GTG CTG was used to detect the WT band at 1,000 bp and reaction 2 with primer pairs AGC CTC TAC ACC CTT CTC TTC TCC ACA and ATC GCC TTC TAT CGC CTT CTT GAC GAG were used to detect KO band at 1,000 bp. For STING, reaction 1 with primer sequences AGA ACG GAC AGC CAG TAA GTA TAC AG and CAA TGC TCT CAT AGC CTT CAC TAT C was used to detect the WT band at 375 bp and reaction 2 with primer pairs AAC TTC CTG ACT AGG GGA GGA GTA G and CAA TGC TCT CAT AGC CTT CAC TAT C was used to detect the KO band at 470 bp.

### Unc93b1 KO mice

*Unc93b1*^−/−^ mice on a C57BL/6 background were developed by K. Fitzgerald at UMass Chan Medical School. The mice were bred and maintained under pathogen-free conditions in our animal facility. The *Unc93b1* deficiency was confirmed by performing PCR on DNA obtained after digesting a tail snip. For *Unc93b1*, specific primer pairs were used to distinguish the WT or KO allele in two separate reactions. Reaction 1 with primer pairs GCA TTC CGA AGC CAC AGC A and TTC GGA GAG TGA CCC TTA TC were used to detect the WT band at 400 bp and reaction 2 with primer pairs GGC TCT TTA CTA TTG CTT TAT G and TTC GGA GAG TGA CCC TTA TC were used to detect KO band at 500 bp.

### Formulations

For PBS (Gibco, catalog no. 10010-023) the base formulation included: 155 mM NaCl, 2.9 mM Na_2_HPO_4_, 1.06 mM KH_2_PO_4_. For aCSF, the base formulation included: 137 mM NaCl, 5 mM KCl, 2.3 mM CaCl_2_, 1.3 mM MgCl_2_, 20 mM glucose, 8 mM HEPES. For LRS, the base formulation included: 100 mM NaCl, 3.7 mM KCl, 1.3 mM CaCl_2_, 1.2 mM MgSO₄, 28 mM NaHCO₃, 20 mM NaC_3_H_5_O_3_ (lactate). All solutions were purchased as *in vivo* grade or filtered sterilized using a 0.2 μm syringe filter (Pall Corporation, NY) prior to *in vivo* delivery. All solutions were physiological pH.

For a 10 mL vial of Elliotts B Solution (buffered intrathecal electrolyte/dextrose injection, Lukare Medical, catalog no. 55792000710), composition was provided on a package insert (as mg/10 mL), which we have converted to molarity as follows for the purposes of comparison: NaCl (73 mg, 125 mM), NaHCO_3_ (19 mg, 23 mM), dextrose (8 mg, 4.4 mM), MgSO_4_·7H_2_O (3 mg, 1.2 mM), KCl (3 mg, 4 mM), CaCl_2_·2H_2_O (2 mg, 1.4 mM), Na_2_PO_4_·7H_2_O (2 mg, 0.75 mM).

### Dynamic light scattering

Buffers described in Table S1 were freshly prepared based on the formulation recipes described above, filtered with a 0.2 μm syringe filter (Pall Corporation, NY) immediately after preparation and kept at room temperature until DLS testing. Elliotts B Solution was purchased at USP grade (Lukare Medical, catalog no. 55792000710) and subjected to the indicated temperature conditions (Table S2). To evaluate dynamic light scattering, 1 mL of each buffer was transferred to a DTS0012 cuvette, and the average size and polydispersity index of any particles detected in the buffers were measured using a Zetasizer Lab (Malvern instruments, MA). Data were obtained in duplicate in each buffer.

### *In vivo* delivery of oligonucleotides to mice

Prior to *in vivo* administration, ASO compounds were resuspended in solution and their concentrations confirmed by UV spectroscopy. Mice were intraperitoneally injected with a fentanyl/midazolam/dexmedetomidine (0.1, 5, and 0.25 mg kg^−1^, respectively, as a solution in sterile saline) cocktail to induce anesthesia. For i.c.v. injections mice received a single bolus injection into the right lateral ventricle (coordinates from bregma reference: −0.4 mm AP, −1.0 mm ML, −2.0 mm DV from skull surface) using a 30-gauge beveled needle. Compounds were injected in a total volume of 10 μL and at a rate of 0.407 μL/s. An intraperitoneal injection of fluemazenil/atipamezole (0.5 and 5 mg kg^−1^, respectively, in sterile saline) was used to reverse injected anesthetic agents. Buprenorphine was also injected for analgesia (0.3 mg kg^−1^, subcutaneously). Animals were removed from the stereotaxic frame and allowed to recover in a warm cage with food and gel provided. EvADINT observation began at the time of anesthetic reversal with animals observed periodically over the next 24 h.

### *In vivo* delivery of oligonucleotides to sheep

We injected *C9ORF72*-targeted ASOs at 2 mg kg^−1^ into sheep by threading a microcatheter up through the intrathecal space and delivering the ASO directly into the cisterna magna, as described.^89^ Intracisternal contrast injection and cone beam computed tomography confirmed the correct catheter position before ASO injection. Two animals received the full PS ASO (Figure 1, sequence 1), while four animals received the mixed backbone ASO (Figure 1, sequence 2). Animals were monitored regularly throughout the first 48 h after injection and any motor phenotypes were noted.

### EvADINT scoring system for acute neurotoxicity

After i.c.v. administration of ASOs to mice, a blinded investigator ranked the behavior of mice at multiple time points using the rubric laid out in Table 1. If a mouse died within the first 24 h, it was automatically assigned a maximum score of 75; otherwise, it was the sum of all other scores. Seizures, if observed, were scored based on severity; hyperactive or spastic behavior was also scored based on severity and included twitching, uncontrolled movement such as “popcorning,” and other atypical motor phenotypes. Besides these behavioral elements, the score was based on the time elapsed until mice resumed normal posture and behavior. For example, if a mouse required >1 h but <2 h to be able to right itself (resume and maintain sternal posture) it would be given a score of 8. Each mouse was individually scored.

### Evaluation of gene silencing in the CNS

#### For comparison of backbones on gene silencing efficacy

Mice were euthanized at 3 weeks post-treatment by cervical dislocation and the brain was immediately removed into ice-cold PBS. The brain was placed in a brain matrix (Braintree Scientific) and the most rostral 3 mm discarded. A 1 mm slice was then taken, and each side homogenized independently. The tissue was suspended in Affymetrix homogenizing solution containing proteinase K and mechanically dissociated using a QIAGEN Tissuelyser with a 2-mm tungsten carbide bead. The tubes were then incubated in a water bath at 65°C until all tubes appeared transparent. Tubes were centrifuged (16,000 × *g*, 15 min) and supernatant transferred to a 96-well plate for storage at −80°C. *Htt, Malat1*, and *Ppib* RNA levels were quantified using the QuantiGene 2.0 assay kit (Affymetrix, QS0011) as described previously.^90^

#### For studying the effect of formulation on gene silencing efficacy

Mice were euthanized at the specified time point post-injection via intraperitoneal administration of 0.1 mL of 390 mg/mL pentobarbital sodium; the brain and spinal cord were immediately removed into ice-cold PBS. Brains were oriented in a stainless-steel brain matrix and coronal sections were collected through the regions of interest; 2-mm biopsy punches were collected from the specific brain areas. Tissue punches were stored in RNAlater overnight at 4°C. Individual punches were homogenized in TRIzol using Fisherbrand Pellet Pestle Cordless Motor and RNase-free disposable pellet pestles (Thermo Fisher Scientific). RNA was extracted using RNA Clean & Concentrator Kit (Zymo) and quantified on a Nanodrop (Thermo Fisher Scientific).

For reverse transcription, 1 μg of RNA was used with the High-Capacity cDNA Reverse Transcription kit (Life Technologies) as per the manufacturer’s protocol. qRT-PCR was carried out in technical duplicates using iTaq Supermix (Bio-Rad) on Bio-Rad CFX-96 real-time machine using gene-specific primers from Integrated DNA Technologies (Coralville, IO): *Malat1* primer 1: 5′ CTC CAA CAA CCA CTA CTC CAA 3′; primer 2: 5′ GTA CTG TTC CAA TCT GCT GCT A 3′; probe: /56-FAM/TCA TAC TCC/ZEN/AGT CGC GTC ACA ATG C/3IABkFQ/. For internal controls: *Ppib* primer 1: 5′ CCG TAG TGC TTC AGC TTG A 3′; primer 2: 5′ AGC AAG TTC CAT CGT GC ATC 3′; probe: /56-FAM/TGC TCT TTC/ZEN/CTC CTG TGC CAT CTC/3IABkFQ/or *Hprt* primer 1: 5′ CCC CAA AAT GGT TAA GGT TGC 3′; primer 2: 5′ AAC AAA GTC TGG CCT GTA TCC 3′; probe: /56-FAM/CTT GCT GGT/ZEN/GAA AAG GAC CTC TCG AA/3IABkFQ/

### Graphs and statistical analyses

Data were analyzed using GraphPad Prism 10.1.2 software for Windows (GraphPad Software, San Diego, CA). For efficacy studies, the levels of mouse RNA from a specific brain region in treated groups were normalized to the mean of the mouse RNA from the corresponding matched region in the vehicle control group. *In vivo* data were analyzed using a one-way ANOVA with a post hoc Tukey multiple comparisons test where *p* values are given and ns, not significant. Graphs are plotted as scatter dot plots (mean ± SEM).

## Data and code availability

Raw mouse neurotoxicity data is presented in the supplemental information. Other raw data can be accessed upon request.

## Acknowledgments

We are grateful to Art Levin for suggesting that we explore the idea of Ca^2+^ formulation as a means of reducing acute neurotoxicity. We thank Nina Bishop, Andrew Coles, Neil Aronin, Miguel Esteves, and Matthew Gounis for help with the sheep study. We thank Anastasia Khvorova for critical feedback on the manuscript. This work was funded by the Ono Pharmaceutical Foundation (Breakthrough Science Award to J.K.W.), the 10.13039/100002108Friedreich's Ataxia Research Alliance (grant to J.K.W.), and the 10.13039/100000002NIH (R01 NS111990 to R.H.B. and J.K.W.). R.H.B. acknowledges funding from The Angel Fund for ALS Research, ALSOne, ALS Finding a Cure, and the Cellucci Fund for ALS Research and the Max Rosenfeld Fund.

## Author contributions

M.P.M., P.M.K., and A. Wagh synthesized and purified oligonucleotides. M.P.M., J.M.R.-B., S.L.S., H.R.M., F.W., M.O., K.K., and A. Weiss contributed experimentally. J.M.R.-B., F.W., and J.K.W. developed the EvADINT scoring assay. M.P.M., J.M.R.-B., M. Marosfoi, R.M.K., and H.G.-E. carried out the sheep study. M. Motwani and K.A.F. developed the KO mice. J.K.W. supervised the study. S.L.S. and J.K.W. wrote the manuscript. All authors edited the manuscript.

## Declaration of interests

F.W., R.H.B., and J.K.W. are co-founders of Nucyrna Therapeutics. J.K.W. is advisory board member of EnPlusOne, PepGen, and Sixfold. R.H.B. is co-founder and Scientific Advisory Board member of ApicBio. The authors have filed patent applications on oligonucleotides and designs intended for use in the CNS.

## Supplemental information

Document S1. Figures S1, S2, and Tables S1–S16

Document S2. Article plus supplemental information
