# Supplementary material for: Quantifying and mitigating motor phenotypes induced by antisense oligonucleotides in the central nervous system
Source: Mol Ther. 2024 Oct 28;32(12):4401–17. doi: 10.1016/j.ymthe.2024.10.024 (PMC11638874; doi:10.1016/j.ymthe.2024.10.024)
Supplement: Document S1. Figures S1, S2, and Tables S1–S16 [file mmc1.pdf]

YMTHE, Volume *32*

Supplemental Information

Quantifying and mitigating motor phenotypes induced by antisense oligonucleotides in the central nervous system

Michael P. Moazami, Julia M. Rembetsy-Brown, Samantha L. Sarli, Holly R. McEachern, Feng Wang, Masahiro Ohara, Atish Wagh, Karen Kelly, Pranathi Meda Krishnamurthy, Alexandra Weiss, Miklos Marosfoi, Robert M. King, Mona Motwani, Heather Gray-Edwards, Katherine A. Fitzgerald, Robert H. Brown, and Jonathan K. Watts

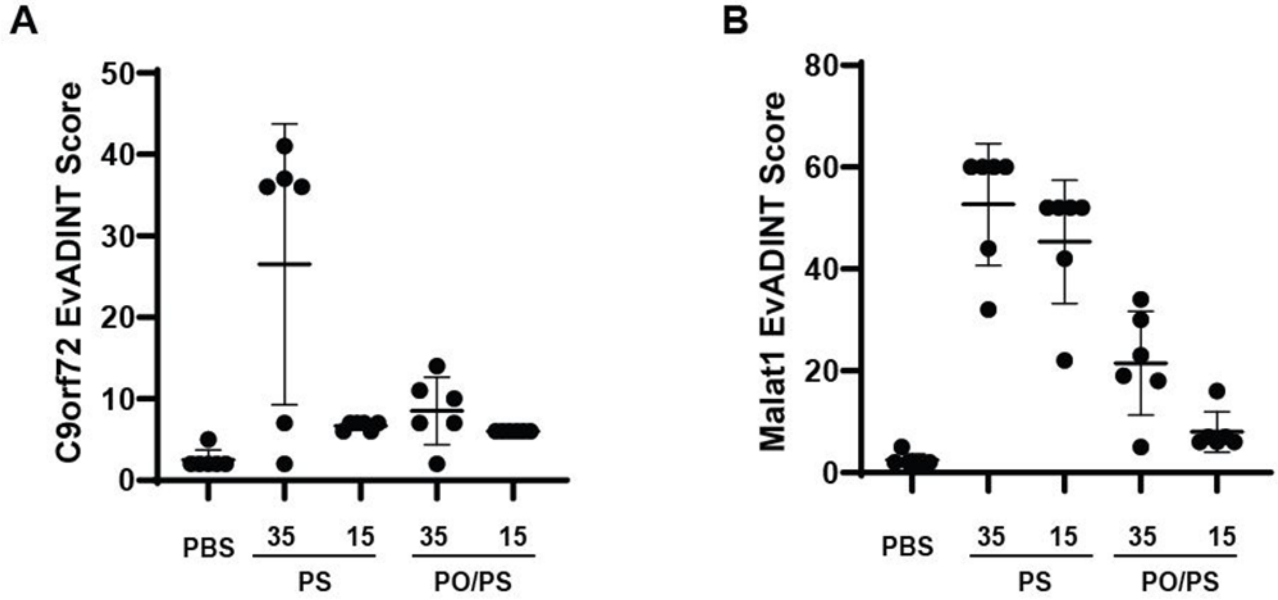

Figure S1. The acute neurotoxicity of ASOs is dose-dependent. Mice were injected ICV with 35 or 15 nmol of ASOs targeting (A) C9orf72 or (B) Malat1 in 10 µL PBS (or with 10 µL PBS as control) and behavior was scored by a blinded investigator over the following 24h using the EvADINT rubric. PS: fully phosphorothioate backbone, PO/PS: mixed backbone. Each data point represents the EvADINT score from one mouse; n = 6; error bars represent SEM.

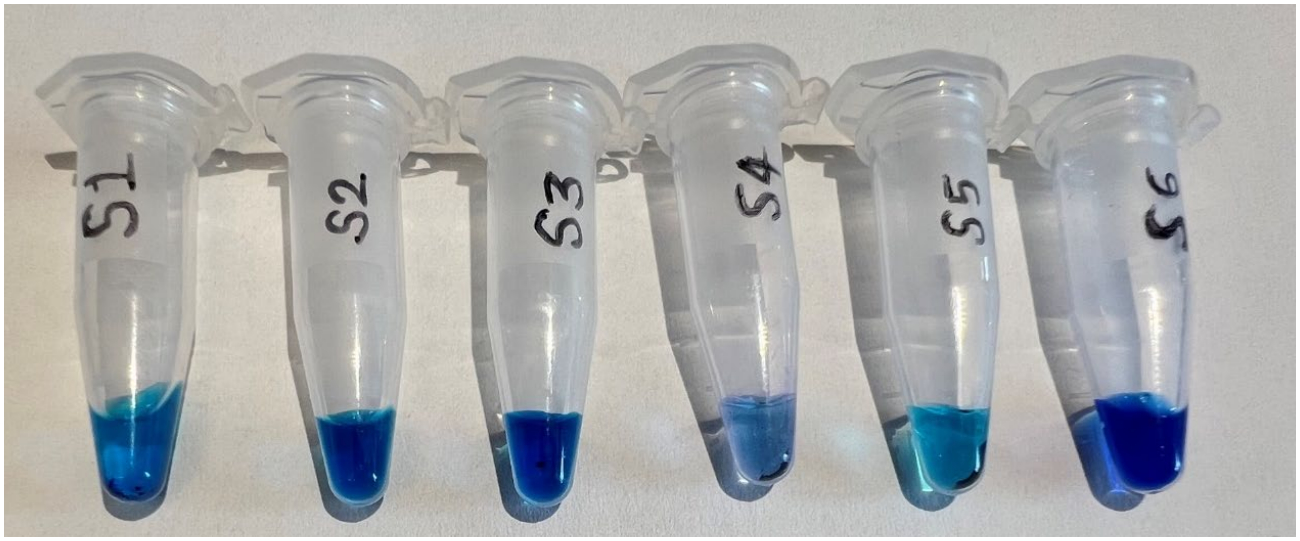

Figure S2. Methylene Blue test for perchlorates

Residual perchlorate ions are highly neurotoxic. To test for the presence of sodium perchlorate after ion-exchange HPLC, we adapted and used a methylene blue test. For this method, ion-exchange HPLC-purified Malat1 ASOs were aliquoted from the same stock and processed using different wash sequences (Kruger, D., and Tschirch, E. (1931). Detection of Small Quantities of Perchlorates. *Z. Anal. Chem* 85, 171-176). We used a 0.3% methylene blue solution, with 5 µL of methylene blue solution added to each 100 µL Malat1 ASO aliquot as well as control standards (0.0001 M, 0.001 or 0.01 M perchlorate ions in tubes S3 to S5, respectively). The mixtures were then centrifuged. In a sample that had undergone insufficient washing (**tube S1**), violet crystalline precipitates were visibly observed indicating trace perchlorates. This was also observed in the three controls spiked with increasing concentrations of sodium perchlorate (**tubes S3-S5**). No precipitates were observed in the ASO sample that was subjected to more rigorous washing (**tube S2**) or the PBS only methylene blue control (**tube S6**).

<u>Sample and observation details</u>

S1: Malat1 Without PBS wash:
We observed a crystalline violet precipitate indicating the presence of perchlorate ions

S2: Malat1 ASO after thorough PBS wash; No precipitate observed.

S3: 0.0001M perchlorate ions;
We observed a slight crystalline violet precipitate indicating the presence of low concentration perchlorate ions

S4: 0.001M perchlorate ions;
We observed a crystalline violet precipitate indicating the presence of perchlorate ions

S5: 0.01M perchlorate ions;
We observed a crystalline violet precipitate indicating the presence of perchlorate ions

S6: PBS only; No precipitate observed.

Table S1. Buffers containing both calcium and phosphate show the presence of micron-scale particles within hours after preparation, even after sterile filtration.

All buffers were filtered using a 0.2 μm syringe filter immediately after preparation. Buffers were tested for the presence of microscopic particles by dynamic light scattering either 3 hours (left) or 24 hours (right) after preparation and sterile filtration of buffer formulations containing divalent cations at the listed concentrations. Solutions were prepared and maintained at room temperature until analysis. All samples were evaluated using DTS0012 cuvettes in duplicate. n.a. = particles were not detected by DLS. PDI = polydispersity index.

	**3 hours**		**24 hours**	
**Formulation**	**Z-Average (d. nm)** mean ± SD	**PdI** mean ± SD	**Z-Average (d. nm)** mean ± SD	**PdI** mean ± SD
PBS (no divalent)	n.a.	n.a.	n.a.	n.a.
PBS +3.6mM Ca^{2+}	**12615 ± 2355**	**0.710 ± 0.257**	**7580 ± 4**	**0.793 ± 0.154**
PBS +3.6mM Mg^{2+}	n.a.	n.a.	n.a.	n.a.
PBS +8mM Ca^{2+}	**11470 ± 594**	**0.793 ± 0.144**	**9210 ± 802**	**0.530 ± 0.204**
PBS +8mM Mg^{2+}	n.a.	n.a.	n.a.	n.a.
aCSF (no divalent)	n.a.	n.a.	n.a.	n.a.
aCSF +3.6mM Ca^{2+}	n.a.	n.a.	n.a.	n.a.
aCSF +3.6mM Mg^{2+}	n.a.	n.a.	n.a.	n.a.
aCSF +8mM Ca^{2+}	n.a.	n.a.	n.a.	n.a.
aCSF +8mM Ca^{2+}	n.a.	n.a.	n.a.	n.a.

Table S2. Particle measurements from Elliotts B solution after heating or freezing.

A USP grade vial of Elliotts B that was stored in 25°C (room temperature) was obtained. 1mL aliquots from this original vial were then briefly heated to 100°C and then stored at room temperature or directly stored at room temperature or -80°C without heating. All solutions were returned to room temperature for analysis. Solutions were tested for the presence of microscopic particles by dynamic light scattering (DLS) (Zetasizer, Malvern Panalytical) using DTS0012 cuvettes in sample duplicates (i.e., sample 1 and sample 2). Each sample duplicate was also measured in technical duplicate (i.e., sample 1.1 and sample 1.2). DLS did not detect any particles from Elliotts B solution stored at room temperature. Perturbations in temperature such as heating to 100°C or even brief storage at -80°C can introduce particles of detectable size by DLS. n.a. = particles were not detected by DLS. PDI = polydispersity index.

Temperature conditions before testing (°C)	Formulation	Z-Average (d. nm)	PdI	Mean Count Rate (kcps)
100	Elliotts B - sample 1.1	**7726**	**0.7705**	**23.05**
100	Elliotts B - sample 1.2	**4879**	**1**	**20.51**
100	Elliotts B - sample 2.1	**14400**	**0.8203**	**14.77**
100	Elliotts B - sample 2.2	**4787**	**1**	**4.033**
25	Elliotts B - sample 3.1	n.a.	n.a.	n.a.
25	Elliotts B - sample 3.2	n.a.	n.a.	n.a.
25	Elliotts B - sample 4.1	n.a.	n.a.	n.a.
25	Elliotts B - sample 4.2	n.a.	n.a.	n.a.
-80	Elliotts B - sample 5.1	n.a.	n.a.	n.a.
-80	Elliotts B - sample 5.2	n.a.	n.a.	n.a.
-80	Elliotts B - sample 6.1	n.a.	n.a.	n.a.
-80	Elliotts B - sample 6.2	**19900**	**1**	**0.429**

Table S3. Raw data from the EvADINT scoring assay for each mouse in Figure 1A

ASO						C9ORF72 Full PS					
Mouse ID						*41*	*42*	*43*	*44*	*45*	*46*
Death			75			0	0	0	0	0	0
	Severe		Moderate		Mild						
Tonic seizure	20		15		10	0	20	15	0	0	15
Hyperactivity or spasticity	15		10		5	5	0	0	10	0	0
Time required for:	0.5 h	1 h	2 h	4 h	≥24 h						
Maintenance of sternal posture	0	4	8	12	20	0	12	8	0	0	12
Unstimulated Movement	0	3	6	9	15	6	15	9	9	3	9
Movement without Ataxia	0	2	4	6	10	6	10	10	4	4	10
Normal Grooming/Eating/Nesting	0	1	2	3	5	3	5	3	3	2	3
Total Score						**20**	**62**	**45**	**26**	**9**	**49**

ASO						C9ORF72 PO/PS					
Mouse ID						*53*	*54*	*55*	*56*	*57*	*58*
Death			75			0	0	0	0	0	0
	Severe		Moderate		Mild						
Tonic seizure	20		15		10	0	0	0	0	0	0
Hyperactivity or spasticity	15		10		5	10	0	0	5	5	0
Time required for:	0.5 h	1 h	2 h	4 h	≥24 h						
Maintenance of sternal posture	0	4	8	12	20	0	0	0	0	8	0
Unstimulated Movement	0	3	6	9	15	9	0	6	6	9	3
Movement without Ataxia	0	2	4	6	10	4	4	6	6	6	4
Normal Grooming/Eating/Nesting	0	1	2	3	5	5	2	2	2	5	1
Total Score						**28**	**6**	**14**	**19**	**33**	**8**

ASO						PBS							
Mouse ID						*33*	*34*	*35*	*36*	*37*	*38*	*39*	*40*
Death			75			0	0	0	0	0	0	0	0
	Severe		Moderate		Mild								
Tonic seizure	20		15		10	0	0	0	0	0	0	0	0
Hyperactivity or spasticity	15		10		5	0	0	0	0	0	0	0	0
Time required for:	0.5 h	1 h	2 h	4 h	≥24 h								
Maintenance of sternal posture	0	4	8	12	20	0	0	0	0	0	0	0	0
Unstimulated Movement	0	3	6	9	15	0	0	0	0	0	0	0	0
Movement without Ataxia	0	2	4	6	10	0	0	0	0	0	0	0	2
Normal Grooming/Eating/Nesting	0	1	2	3	5	1	1	0	1	1	1	1	0
Total Score						**1**	**1**	**0**	**1**	**1**	**1**	**1**	**2**

Table S4. Raw data from the EvADINT scoring assay for each mouse in Figure 1B

ASO						Malat1 Full PS					
**Mouse ID**						***105***	***106***	***107***	***108***	***109***	***110***
**Death**			75			0	0	0	75	0	0
	**Severe**		**Moderate**		**Mild**						
**Tonic seizure**	20		15		10	15	0	0	0	10	10
**Hyperactivity or spasticity**	15		10		5	0	10	10	0	0	0
**Time required for:**	**0.5 h**	**1 h**	**2 h**	**4 h**	**≥24 h**						
**Maintenance of sternal posture**	0	4	8	12	20	20	0	20	0	20	20
**Unstimulated Movement**	0	3	6	9	15	15	3	15	0	15	15
**Movement without Ataxia**	0	2	4	6	10	10	6	10	0	10	10
**Normal Grooming/Eating/Nesting**	0	1	2	3	5	5	1	5	0	5	5
**Total Score**						**65**	**20**	**60**	**75**	**60**	**60**

ASO						Malat1 PO/PS					
**Mouse ID**						***117***	***118***	***119***	***120***	***121***	***122***
**Death**			75			0	0	0	0	0	0
	**Severe**		**Moderate**		**Mild**						
**Tonic seizure**	20		15		10	0	0	0	0	0	0
**Hyperactivity or spasticity**	15		10		5	5	5	5	5	0	0
**Time required for:**	**0.5 h**	**1 h**	**2 h**	**4 h**	**≥24 h**						
**Maintenance of sternal posture**	0	4	8	12	20	12	12	12	12	8	8
**Unstimulated Movement**	0	3	6	9	15	9	9	9	9	6	9
**Movement without Ataxia**	0	2	4	6	10	10	10	10	10	10	10
**Normal Grooming/Eating/Nesting**	0	1	2	3	5	3	5	3	5	2	3
**Total Score**						**39**	**41**	**39**	**41**	**26**	**30**

ASO						PBS					
**Mouse ID**						***129***	***130***	***131***	***132***	***133***	***134***
**Death**			75			0	0	0	0	0	0
	**Severe**		**Moderate**		**Mild**						
**Tonic seizure**	20		15		10	0	0	0	0	0	0
**Hyperactivity or spasticity**	15		10		5	0	0	0	0	0	0
**Time required for:**	**0.5 h**	**1 h**	**2 h**	**4 h**	**≥24 h**						
**Maintenance of sternal posture**	0	4	8	12	20	0	0	0	0	0	0
**Unstimulated Movement**	0	3	6	9	15	0	0	0	0	0	0
**Movement without Ataxia**	0	2	4	6	10	2	2	2	2	2	2
**Normal Grooming/Eating/Nesting**	0	1	2	3	5	1	1	1	1	1	1
**Total Score**						**3**	**3**	**3**	**3**	**3**	**3**

Table S5. Raw data from the EvADINT scoring assay for each mouse in Figure 1C

ASO						Htt Full PS							
**Mouse ID**						***1***	***2***	***3***	***4***	***5***	***6***	***7***	***8***
**Death**			75			0	0	0	0	0	0	0	0
	**Severe**		**Moderate**		**Mild**								
**Tonic seizure**	20		15		10	0	0	0	0	15	0	0	15
**Hyperactivity or spasticity**	15		10		5	0	10	10	10	0	0	10	0
**Time required for:**	**0.5 h**	**1 h**	**2 h**	**4 h**	**≥24 h**								
**Maintenance of sternal posture**	0	4	8	12	20	0	0	0	0	20	12	4	20
**Unstimulated Movement**	0	3	6	9	15	9	9	9	9	15	9	6	15
**Movement without Ataxia**	0	2	4	6	10	6	10	10	10	10	3	6	10
**Normal Grooming/Eating/Nesting**	0	1	2	3	5	5	3	3	3	5	3	2	5
**Total Score**						**20**	**32**	**32**	**32**	**65**	**27**	**28**	**65**

ASO						Htt PO/PS							
**Mouse ID**						***17***	***18***	***19***	***20***	***21***	***22***	***23***	***24***
**Death**			75			0	75	0	0	0	0	0	
	**Severe**		**Moderate**		**Mild**								
**Tonic seizure**	20		15		10	0	0	0	0	0	20	0	0
**Hyperactivity or spasticity**	15		10		5	0	0	0	0	0	0	15	5
**Time required for:**	**0.5 h**	**1 h**	**2 h**	**4 h**	**≥24 h**								
**Maintenance of sternal posture**	0	4	8	12	20	0	0	0	0	0	12	4	0
**Unstimulated Movement**	0	3	6	9	15	6	0	0	9	0	15	9	3
**Movement without Ataxia**	0	2	4	6	10	6	0	4	10	4	10	6	4
**Normal Grooming/Eating/Nesting**	0	1	2	3	5	5	0	3	1	0	5	3	2
**Total Score**						**17**	**75**	**7**	**20**	**4**	**62**	**37**	**14**

ASO						PBS							
**Mouse ID**						***33***	***34***	***35***	***36***	***37***	***38***	***39***	***40***
**Death**			75			0	0	0	0	0	0	0	0
	**Severe**		**Moderate**		**Mild**								
**Tonic seizure**	20		15		10	0	0	0	0	0	0	0	0
**Hyperactivity or spasticity**	15		10		5	0	0	0	0	0	0	0	0
**Time required for:**	**0.5 h**	**1 h**	**2 h**	**4 h**	**≥24 h**								
**Maintenance of sternal posture**	0	4	8	12	20	0	0	0	0	0	0	0	0
**Unstimulated Movement**	0	3	6	9	15	0	0	0	0	0	0	0	0
**Movement without Ataxia**	0	2	4	6	10	0	0	0	0	0	0	0	2
**Normal Grooming/Eating/Nesting**	0	1	2	3	5	1	1	0	1	1	1	1	0
**Total Score**						**1**	**1**	**0**	**1**	**1**	**1**	**1**	**2**

Table S6. Raw data from the EvADINT scoring assay for each mouse in Figure 1D

**ASO**						**NTC Full PS**					
**Mouse ID**						***135***	***136***	***137***	***138***	***139***	***140***
**Death**			75			75	0	0	0	0	0
	**Severe**		**Moderate**		**Mild**						
**Tonic seizure**	20		15		10	0	0	0	0	0	0
**Hyperactivity or spasticity**	15		10		5	0	10	10	10	10	5
**Time required for:**	**0.5 h**	**1 h**	**2 h**	**4 h**	**≥24 h**						
**Maintenance of sternal posture**	0	4	8	12	20	0	20	20	0	20	0
**Unstimulated Movement**	0	3	6	9	15	0	15	15	9	15	3
**Movement without Ataxia**	0	2	4	6	10	0	10	10	6	10	6
**Normal Grooming/Eating/Nesting**	0	1	2	3	5	0	5	5	3	5	1
**Total Score**						**75**	**60**	**60**	**28**	**60**	**15**

**ASO**						**NTC PO/PS**					
**Mouse ID**						***147***	***148***	***149***	***150***	***151***	***152***
**Death**			75			0	0	0	0	0	0
	**Severe**		**Moderate**		**Mild**						
**Tonic seizure**	20		15		10	0	0	0	0	0	0
**Hyperactivity or spasticity**	15		10		5	0	0	0	0	0	0
**Time required for:**	**0.5 h**	**1 h**	**2 h**	**4 h**	**≥24 h**						
**Maintenance of sternal posture**	0	4	8	12	20	0	0	0	0	0	0
**Unstimulated Movement**	0	3	6	9	15	0	0	0	0	0	0
**Movement without Ataxia**	0	2	4	6	10	4	4	4	4	4	4
**Normal Grooming/Eating/Nesting**	0	1	2	3	5	2	2	2	2	0	1
**Total Score**						**6**	**6**	**6**	**6**	**4**	**5**

**ASO**						**PBS**					
**Mouse ID**						***129***	***130***	***131***	***132***	***133***	***134***
**Death**			75			0	0	0	0	0	0
	**Severe**		**Moderate**		**Mild**						
**Tonic seizure**	20		15		10	0	0	0	0	0	0
**Hyperactivity or spasticity**	15		10		5	0	0	0	0	0	0
**Time required for:**	**0.5 h**	**1 h**	**2 h**	**4 h**	**≥24 h**						
**Maintenance of sternal posture**	0	4	8	12	20	0	0	0	0	0	0
**Unstimulated Movement**	0	3	6	9	15	0	0	0	0	0	0
**Movement without Ataxia**	0	2	4	6	10	2	2	2	2	2	2
**Normal Grooming/Eating/Nesting**	0	1	2	3	5	1	1	1	1	1	1
**Total Score**						**3**	**3**	**3**	**3**	**3**	**3**

Table S7. Raw data from the EvADINT scoring assay for each mouse in Figure 2

ASO						PS DNA							
**Mouse ID**						***65***	***66***	***67***	***68***	***69***	***70***	***71***	***72***
**Death**			75			0	0	0	0	0	0	0	75
	**Severe**		**Moderate**		**Mild**								
**Tonic seizure**	20		15		10	20	0	20	20	20	15	10	0
**Hyperactivity or spasticity**	15		10		5	0	0	0	0	0	0	0	0
**Time required for:**	**0.5 h**	**1 h**	**2 h**	**4 h**	**≥24 h**								
**Maintenance of sternal posture**	0	4	8	12	20	20	0	20	20	20	4	4	0
**Unstimulated Movement**	0	3	6	9	15	15	9	15	15	15	6	15	0
**Movement without Ataxia**	0	2	4	6	10	10	10	10	10	10	6	10	0
**Normal Grooming/Eating/Nesting**	0	1	2	3	5	5	5	5	5	5	5	5	0
**Total Score**						**70**	**24**	**70**	**70**	**70**	**36**	**44**	**75**

ASO						PS 2'-O-MOE							
**Mouse ID**						***73***	***74***	***75***	***76***	***77***	***78***	***79***	***80***
**Death**			75			0	0	0	0	0	0	0	0
	**Severe**		**Moderate**		**Mild**								
**Tonic seizure**	20		15		10	0	0	0	0	0	10	10	0
**Hyperactivity or spasticity**	15		10		5	10	10	10	10	10	0	0	10
**Time required for:**	**0.5 h**	**1 h**	**2 h**	**4 h**	**≥24 h**								
**Maintenance of sternal posture**	0	4	8	12	20	4	4	4	4	4	0	0	4
**Unstimulated Movement**	0	3	6	9	15	9	9	9	6	9	0	0	9
**Movement without Ataxia**	0	2	4	6	10	6	6	6	6	6	6	6	6
**Normal Grooming/Eating/Nesting**	0	1	2	3	5	3	3	3	3	3	3	3	3
**Total Score**						**32**	**32**	**32**	**29**	**32**	**19**	**19**	**32**

ASO						PS 2'-O-Me							
**Mouse ID**						***81***	***82***	***83***	***84***	***85***	***86***	***87***	***88***
**Death**			75			0	0	0	0	0	0	0	0
	**Severe**		**Moderate**		**Mild**								
**Tonic seizure**	20		15		10	0	0	0	0	0	0	0	10
**Hyperactivity or spasticity**	15		10		5	5	10	10	15	10	5	5	0
**Time required for:**	**0.5 h**	**1 h**	**2 h**	**4 h**	**≥24 h**								
**Maintenance of sternal posture**	0	4	8	12	20	8	4	0	8	4	0	0	0
**Unstimulated Movement**	0	3	6	9	15	6	3	3	9	3	0	3	6
**Movement without Ataxia**	0	2	4	6	10	6	6	4	6	6	6	6	10
**Normal Grooming/Eating/Nesting**	0	1	2	3	5	3	3	3	3	3	5	3	3
**Total Score**						**28**	**26**	**20**	**41**	**26**	**16**	**17**	**29**

**ASO**									**PBS**				
**Mouse ID**						***89***	***90***	***91***	***92***	***93***	***94***	***95***	***96***
**Death**			75			0	0	0	0	0	0	0	0
	**Severe**		**Moderate**		**Mild**								
**Tonic seizure**	20		15		10	0	0	0	0	0	0	0	0
**Hyperactivity or spasticity**	15		10		5	0	0	5	0	0	0	0	0
**Time required for:**	**0.5 h**	**1 h**	**2 h**	**4 h**	**≥24 h**								
**Maintenance of sternal posture**	0	4	8	12	20	0	0	0	0	0	0	0	0
**Unstimulated Movement**	0	3	6	9	15	0	0	0	0	0	0	0	0
**Movement without Ataxia**	0	2	4	6	10	2	2	4	4	4	2	2	4
**Normal Grooming/Eating/Nesting**	0	1	2	3	5	0	0	1	0	0	0	0	0
**Total Score**						**2**	**2**	**10**	**4**	**4**	**2**	**2**	**4**

Table S8. Raw data from the EvADINT scoring assay for each mouse in Figure 3a

ASO						B6 WT / PBS		
**Mouse ID**						***6769***	***6770***	***6751***
**Death**			75			0	0	0
		**Severe**		**Moderate**	**Mild**			
**Tonic seizure**		20		15	10	0	0	0
**Hyperactivity or spasticity**		15		10	5	0	0	0
**Time required for:**	**0.5 h**	**1 h**	**2 h**	**4 h**	**≥24 h**			
**Maintenance of sternal posture**	0	4	8	12	20	0	0	0
**Unstimulated Movement**	0	3	6	9	15	0	0	0
**Movement without Ataxia**	0	2	4	6	10	0	0	0
**Normal Grooming/Eating/Nesting**	0	1	2	3	5	1	1	1
**Total Score**						1	1	1

ASO						UNC93B1 / PBS		
**Mouse ID**						***6752***	***6753***	***6754***
**Death**			75			0	0	0
		**Severe**		**Moderate**	**Mild**			
**Tonic seizure**		20		15	10	0	0	0
**Hyperactivity or spasticity**		15		10	5	0	0	0
**Time required for:**	**0.5 h**	**1 h**	**2 h**	**4 h**	**≥24 h**			
**Maintenance of sternal posture**	0	4	8	12	20	0	0	0
**Unstimulated Movement**	0	3	6	9	15	0	0	0
**Movement without Ataxia**	0	2	4	6	10	2	2	2
**Normal Grooming/Eating/Nesting**	0	1	2	3	5	2	2	2
**Total Score**						4	4	4

ASO						B6 WT / Full DNA		
**Mouse ID**						***6758***	***6759***	***6760***
**Death**			75			75	75	75
		**Severe**		**Moderate**	**Mild**			
**Tonic seizure**		20		15	10	0	0	0
**Hyperactivity or spasticity**		15		10	5	0	0	0
**Time required for:**	**0.5 h**	**1 h**	**2 h**	**4 h**	**≥24 h**			
**Maintenance of sternal posture**	0	4	8	12	20	0	0	0
**Unstimulated Movement**	0	3	6	9	15	0	0	0
**Movement without Ataxia**	0	2	4	6	10	0	0	0
**Normal Grooming/Eating/Nesting**	0	1	2	3	5	0	0	0
**Total Score**						75	75	75

**ASO**									**UNC93B1 / Full DNA**		
**Mouse ID**									***6755***	***6756***	***6757***
**Death**				75					75	75	75
		**Severe**				**Moderate**		**Mild**			
**Tonic seizure**		20				15		10	0	0	0
**Hyperactivity or spasticity**		15				10		5	0	0	0
**Time required for:**	**0.5 h**		**1 h**		**2 h**		**4 h**	**≥24 h**			
**Maintenance of sternal posture**	0		4		8		12	20	0	0	0
**Unstimulated Movement**	0		3		6		9	15	0	0	0
**Movement without Ataxia**	0		2		4		6	10	0	0	0
**Normal Grooming/Eating/Nesting**	0		1		2		3	5	0	0	0
**Total Score**									75	75	75

Table S9. Raw data from the EvADINT scoring assay for each mouse in Figure 3b

ASO						B6 WT / PBS				
Mouse ID						*159*	*160*	*161*	*162*	*163*
Death			75			0	0	0	0	0
	Severe		Moderate		Mild					
Tonic seizure	20		15		10	0	0	0	0	0
Hyperactivity or spasticity	15		10		5	0	0	0	0	0
Time required for:	0.5 h	1 h	2 h	4 h	≥24 h					
Maintenance of sternal posture	0	4	8	12	20	0	0	0	0	0
Unstimulated Movement	0	3	6	9	15	0	0	0	0	0
Movement without Ataxia	0	2	4	6	10	4	4	4	4	4
Normal Grooming/Eating/Nesting	0	1	2	3	5	2	2	2	2	2
Total Score						**6**	**6**	**6**	**6**	**6**

ASO						B6 WT / Full DNA				
Mouse ID						*164*	*165*	*166*	*167*	*168*
Death			75			75	0	75	0	75
	Severe		Moderate		Mild					
Tonic seizure	20		15		10	0	0	0	10	0
Hyperactivity or spasticity	15		10		5	0	15	0	0	0
Time required for:	0.5 h	1 h	2 h	4 h	≥24 h					
Maintenance of sternal posture	0	4	8	12	20	0	20	0	20	0
Unstimulated Movement	0	3	6	9	15	0	15	0	15	0
Movement without Ataxia	0	2	4	6	10	0	10	0	10	0
Normal Grooming/Eating/Nesting	0	1	2	3	5	0	5	0	5	0
Total Score						**75**	**65**	**75**	**60**	**75**

ASO						MyD88.STING / PBS		
Mouse ID						*169*	*170*	*171*
Death			75			0	0	0
	Severe		Moderate		Mild			
Tonic seizure	20		15		10	0	0	0
Hyperactivity or spasticity	15		10		5	0	0	0
Time required for:	0.5 h	1 h	2 h	4 h	≥24 h			
Maintenance of sternal posture	0	4	8	12	20	0	0	0
Unstimulated Movement	0	3	6	9	15	0	0	0
Movement without Ataxia	0	2	4	6	10	4	4	4
Normal Grooming/Eating/Nesting	0	1	2	3	5	1	2	1
Total Score						**5**	**6**	**5**

**ASO**						**MyD88.Sting / Full DNA**			
**Mouse ID**						***172***	***173***	***174***	***175***
**Death**			75			75	75	75	0
	**Severe**		**Moderate**		**Mild**				
**Seizure**	20		15		10	0	0	0	10
**Hyperactivity or spasms**	15		10		5	0	0	0	0
**Time required for:**	**0.5 h**	**1 h**	**2 h**	**4 h**	**≥24 h**				
**Maintenance of sternal posture**	0	4	8	12	20	0	0	0	20
**Unstimulated Movement**	0	3	6	9	15	0	0	0	15
**Movement without Ataxia**	0	2	4	6	10	0	0	0	10
**Normal Grooming/Eating/Nesting**	0	1	2	3	5	0	0	0	5
**Total Score**						**75**	**75**	**75**	**60**

Table S10. Raw data from the EvADINT scoring assay for each mouse in Figure 4a

ASO						C9ORF72 Full PS w/Ca2+					
**Mouse ID**						***47***	***48***	***49***	***50***	***51***	***52***
**Death**			75			0	0	0	0	0	0
	**Severe**		**Moderate**		**Mild**						
**Tonic seizure**	20		15		10	0	0	0	0	0	0
**Hyperactivity or spasticity**	15		10		5	10	0	5	5	0	0
**Time required for:**	**0.5 h**	**1 h**	**2 h**	**4 h**	**≥24 h**						
**Maintenance of sternal posture**	0	4	8	12	20	8	0	12	8	0	12
**Unstimulated Movement**	0	3	6	9	15	9	0	9	9	0	9
**Movement without Ataxia**	0	2	4	6	10	6	4	10	10	2	10
**Normal Grooming/Eating/Nesting**	0	1	2	3	5	3	3	5	5	0	5
**Total Score**						**36**	**7**	**41**	**37**	**2**	**36**

ASO						C9ORF72 PO/PS w/Ca2+					
**Mouse ID**						***59***	***60***	***61***	***62***	***63***	***64***
**Death**			75			0	0	0	0	0	0
	**Severe**		**Moderate**		**Mild**						
**Tonic seizure**	20		15		10	0	0	0	0	0	0
**Hyperactivity or spasticity**	15		10		5	0	0	0	0	0	0
**Time required for:**	**0.5 h**	**1 h**	**2 h**	**4 h**	**≥24 h**						
**Maintenance of sternal posture**	0	4	8	12	20	0	0	0	0	0	0
**Unstimulated Movement**	0	3	6	9	15	0	3	3	6	0	0
**Movement without Ataxia**	0	2	4	6	10	0	6	6	6	6	6
**Normal Grooming/Eating/Nesting**	0	1	2	3	5	2	1	2	2	1	1
**Total Score**						**2**	**10**	**11**	**14**	**7**	**7**

Table S11. Raw data from the EvADINT scoring assay for each mouse in Figure 4b

**ASO**						Malat1 Full PS w/Ca2+					
**Mouse ID**						***111***	***112***	***113***	***114***	***115***	***116***
**Death**			75			0	0	0	0	0	0
	**Severe**		**Moderate**		**Mild**						
**Tonic seizure**	20		15		10	10	10	10	10	10	10
**Hyperactivity or spasticity**	15		10		5	0	0	0	0	0	0
**Time required for:**	**0.5 h**	**1 h**	**2 h**	**4 h**	**≥24 h**						
**Maintenance of sternal posture**	0	4	8	12	20	12	20	0	20	20	20
**Unstimulated Movement**	0	3	6	9	15	9	15	9	15	15	15
**Movement without Ataxia**	0	2	4	6	10	10	10	10	10	10	10
**Normal Grooming/Eating/Nesting**	0	1	2	3	5	3	5	3	5	5	5
**Total Score**						**44**	**60**	**32**	**60**	**60**	**60**

**ASO**						Malat1 PO/PS w/Ca2+					
**Mouse ID**						***123***	***124***	***125***	***126***	***127***	***128***
**Death**			75			0	0	0	0	0	0
	**Severe**		**Moderate**		**Mild**						
**Tonic seizure**	20		15		10	0	0	0	0	0	0
**Hyperactivity or spasticity**	15		10		5	5	0	0	0	0	0
**Time required for:**	**0.5 h**	**1 h**	**2 h**	**4 h**	**≥24 h**						
**Maintenance of sternal posture**	0	4	8	12	20	0	0	8	0	0	12
**Unstimulated Movement**	0	3	6	9	15	9	6	9	6	0	9
**Movement without Ataxia**	0	2	4	6	10	6	10	10	10	4	10
**Normal Grooming/Eating/Nesting**	0	1	2	3	5	3	2	3	3	1	3
**Total Score**						**23**	**18**	**30**	**19**	**5**	**34**

Table S12. Raw data from the EvADINT scoring assay for each mouse in Figure 4c

**ASO**									**Htt Full PS w/Ca2+**				
**Mouse ID**						***9***	***10***	***11***	***12***	***13***	***14***	***15***	***16***
**Death**			75			0	0	0	0	0	0	0	0
	**Severe**		**Moderate**		**Mild**								
**Tonic seizure**	20		15		10	0	0	0	0	0	0	0	0
**Hyperactivity or spasticity**	15		10		5	0	0	0	0	0	0	0	0
**Time required for:**	**0.5 h**	**1 h**	**2 h**	**4 h**	**≥24 h**								
**Maintenance of sternal posture**	0	4	8	12	20	0	4	0	0	0	0	0	0
**Unstimulated Movement**	0	3	6	9	15	0	9	0	3	6	3	6	3
**Movement without Ataxia**	0	2	4	6	10	0	10	10	10	10	6	2	4
**Normal Grooming/Eating/Nesting**	0	1	2	3	5	3	5	0	1	2	2	6	2
**Total Score**						**3**	**28**	**10**	**14**	**18**	**11**	**14**	**9**

**ASO**									**Htt PO/PS w/Ca2+**				
**Mouse ID**						***25***	***26***	***27***	***28***	***29***	***30***	***31***	***32***
**Death**			75			0	0	0	75	0	0	0	0
	**Severe**		**Moderate**		**Mild**								
**Tonic seizure**	20		15		10	0	0	0	0	0	0	0	0
**Hyperactivity or spasticity**	15		10		5	0	0	0	0	0	0	0	0
**Time required for:**	**0.5 h**	**1 h**	**2 h**	**4 h**	**≥24 h**								
**Maintenance of sternal posture**	0	4	8	12	20	0	0	0	0	0	0	0	0
**Unstimulated Movement**	0	3	6	9	15	3	3	6	0	3	0	0	0
**Movement without Ataxia**	0	2	4	6	10	6	10	10	0	10	2	2	2
**Normal Grooming/Eating/Nesting**	0	1	2	3	5	3	2	2	0	1	0	0	0
**Total Score**						**12**	**15**	**18**	**75**	**14**	**2**	**2**	**2**

Table S13. Raw data from the EvADINT scoring assay for each mouse in Figure 4d

ASO						NTC Full PS w/Ca2+					
**Mouse ID**						***141***	***142***	***143***	***144***	***145***	***146***
**Death**			75			0	0	0	0	0	75
	**Severe**		**Moderate**		**Mild**						
**Tonic seizure**	20		15		10	0	0	0	0	0	0
**Hyperactivity or spasticity**	15		10		5	10	10	10	10	10	0
**Time required for:**	**0.5 h**	**1 h**	**2 h**	**4 h**	**≥24 h**						
**Maintenance of sternal posture**	0	4	8	12	20	12	12	12	12	12	0
**Unstimulated Movement**	0	3	6	9	15	15	15	9	9	9	0
**Movement without Ataxia**	0	2	4	6	10	10	10	10	10	10	0
**Normal Grooming/Eating/Nesting**	0	1	2	3	5	5	5	5	5	3	0
**Total Score**						**0**	**52**	**46**	**46**	**44**	**75**

ASO						NTC PO/PS w/Ca2+					
**Mouse ID**						***153***	***154***	***155***	***156***	***157***	***158***
**Death**			75			0	0	0	0	0	0
	**Severe**		**Moderate**		**Mild**						
**Tonic seizure**	20		15		10	0	0	0	0	0	0
**Hyperactivity or spasticity**	15		10		5	0	0	0	0	0	0
**Time required for:**	**0.5 h**	**1 h**	**2 h**	**4 h**	**≥24 h**						
**Maintenance of sternal posture**	0	4	8	12	20	0	0	0	0	0	0
**Unstimulated Movement**	0	3	6	9	15	0	0	0	0	0	0
**Movement without Ataxia**	0	2	4	6	10	4	4	4	4	4	4
**Normal Grooming/Eating/Nesting**	0	1	2	3	5	0	0	1	2	1	1
**Total Score**						**4**	**4**	**5**	**6**	**5**	**5**

Table S14. Raw data from the EvADINT scoring assay for each mouse in Figure 5. Part 1/7

ASO						PBS									
Mouse ID						*8249*	*8250*	*8253*	*8256*	*8259*	*8931*	*8932*	*8133*	*8134*	*8135*
Death			75			0	0	0	0	0	0	0	0	0	0
	**Severe**		**Moderate**		**Mild**										
Tonic seizure	20		15		10	0	0	0	0	0	0	0	0	0	0
Hyperactivity or spasticity	15		10		5	0	0	0	0	0	0	0	0	0	0
Time required for:	0.5 h	1 h	2 h	4 h	≥24 h										
Maintenance of sternal posture	0	4	8	12	20	8	0	0	12	8	0	12	0	12	0
Unstimulated Movement	0	3	6	9	15	6	0	0	9	6	0	9	0	9	0
Movement without Ataxia	0	2	4	6	10	6	6	6	6	6	4	10	2	6	4
Normal Grooming/Eating/Nesting	0	1	2	3	5	3	3	1	5	3	3	5	5	3	3
Total Score						**23**	**9**	**7**	**32**	**23**	**7**	**36**	**7**	**30**	**7**

ASO						PBS (8Ca)				
Mouse ID						*8246*	*8247*	*8252*	*8255*	*8258*
Death			75			0	0	0	0	0
	**Severe**		**Moderate**		**Mild**					
Tonic seizure	20		15		10	0	0	0	0	0
Hyperactivity or spasticity	15		10		5	0	0	0	0	0
Time required for:	0.5 h	1 h	2 h	4 h	≥24 h					
Maintenance of sternal posture	0	4	8	12	20	0	12	12	12	20
Unstimulated Movement	0	3	6	9	15	6	9	6	6	15
Movement without Ataxia	0	2	4	6	10	4	6	6	6	10
Normal Grooming/Eating/Nesting	0	1	2	3	5	3	3	5	5	5
Total Score						**13**	**30**	**29**	**29**	**50**

Table S14 continued (part 2/7).

ASO						PBS (8Mg)									
Mouse ID						*8236*	*8239*	*8240*	*8242*	*8243*	*8902*	*8905*	*8908*	*8911*	*8914*
Death			75			0	0	0	0	0	0	0	0	0	0
	Severe		Moderate		Mild										
Tonic seizure	20		15		10	0	10	0	0	0	0	0	0	0	0
Hyperactivity or spasticity	15		10		5	0	0	0	0	0	0	0	0	0	0
Time required for:	0.5 h	1 h	2 h	4 h	≥24 h										
Maintenance of sternal posture	0	4	8	12	20	8	0	0	4	20	0	0	0	0	0
Unstimulated Movement	0	3	6	9	15	6	0	0	6	15	0		0	0	3
Movement without Ataxia	0	2	4	6	10	6	6	6	6	10	2	0	2	0	2
Normal Grooming/Eating/Nesting	0	1	2	3	5	5	5	3	3	5	2	5	3	3	2
Total Score						**25**	**21**	**9**	**19**	**50**	**4**	**5**	**5**	**3**	**7**

ASO						PBS (3.6Mg)				
Mouse ID						*8939*	*8941*	*8944*	*8947*	*8950*
Death			75			0	0	0	0	0
	Severe		Moderate		Mild					
Tonic seizure	20		15		10	0	0	0	0	0
Hyperactivity or spasticity	15		10		5	0	0	0	0	0
Time required for:	0.5 h	1 h	2 h	4 h	≥24 h					
Maintenance of sternal posture	0	4	8	12	20	0	8	8	4	0
Unstimulated Movement	0	3	6	9	15	0	6	6	3	0
Movement without Ataxia	0	2	4	6	10	2	6	6	4	6
Normal Grooming/Eating/Nesting	0	1	2	3	5	2	5	5	3	3
Total Score						**4**	**25**	**25**	**14**	**9**

Table S14 continued (part 3/7).

ASO						PBS (empty)									
Mouse ID						*8261*	*8262*	*8263*	*8264*	*8265*	*8936*	*8939*	*8942*	*8945*	*8948*
Death			75			0	0	0	0	0	0	0	0	0	0
	Severe		Moderate		Mild										
Tonic seizure	20		15		10	0	0	0	0	0	0	0	0	0	0
Hyperactivity or spasticity	15		10		5	0	0	0	0	0	0	0	0	0	0
Time required for:	0.5 h	1 h	2 h	4 h	≥24 h										
Maintenance of sternal posture	0	4	8	12	20	0	0	0	0	0	0	0	0	0	0
Unstimulated Movement	0	3	6	9	15	0	0	0	0	0	0	0	0	0	0
Movement without Ataxia	0	2	4	6	10	0	0	0	0	0	2	0	0	0	0
Normal Grooming/Eating/Nesting	0	1	2	3	5	1	0	1	0	0	1	1	1	0	1
Total Score						**1**	**0**	**1**	**0**	**0**	**3**	**1**	**1**	**0**	**1**

ASO						aCSF									
Mouse ID						*8201*	*8204*	*8207*	*8210*	*8213*	*8903*	*8906*	*8909*	*8912*	*8915*
Death			75			0	0	0	0	0	0	0	0	0	0
	Severe		Moderate		Mild										
Tonic seizure	20		15		10	0	0	0	0	0	0	0	0	0	0
Hyperactivity or spasticity	15		10		5	0	0	0	0	0	0	0	0	0	0
Time required for:	0.5 h	1 h	2 h	4 h	≥24 h										
Maintenance of sternal posture	0	4	8	12	20	0	0	0	0	0	0	0	4	0	4
Unstimulated Movement	0	3	6	9	15	0	0	0	2	0	0	0	3	3	6
Movement without Ataxia	0	2	4	6	10	2	0	0	0	0	0	0	4	2	6
Normal Grooming/Eating/Nesting	0	1	2	3	5	5	5	3	5	3	1	2	3	5	3
Total Score						**7**	**5**	**3**	**7**	**3**	**1**	**2**	**14**	**10**	**19**

Table S14 continued (part 4/7).

ASO						aCSF (8Ca)									
Mouse ID						***8221***	***8224***	***8227***	***8230***	***8233***	***8937***	***8940***	***8943***	***8946***	***8949***
Death			75			0	0	0	0	0	0	0	0	0	0
	**Severe**		**Moderate**		**Mild**										
Tonic seizure	20		15		10	10	0	0	0	10	0	0	0	0	0
Hyperactivity or spasticity	15		10		5	0	0	0	0	0	0	0	0	0	0
Time required for:	**0.5 h**	**1 h**	**2 h**	**4 h**	**≥24 h**										
Maintenance of sternal posture	0	4	8	12	20	4	0	0	0	0	0	8	8	0	0
Unstimulated Movement	0	3	6	9	15	0	0	0	0	0	0	6	6	0	6
Movement without Ataxia	0	2	4	6	10	4	0	0	0	2	2	6	4	6	6
Normal Grooming/Eating/Nesting	0	1	2	3	5	3	3	2	3	2	2	3	3	5	3
Total Score						**21**	**3**	**2**	**3**	**14**	**4**	**23**	**21**	**11**	**15**

ASO						aCSF (8Mg)									
Mouse ID						***8222***	***8225***	***8228***	***8231***	***8234***	***8901***	***8904***	***8907***	***8910***	***8913***
Death			75			0	0	0	0	0	0	0	0	0	0
	**Severe**		**Moderate**		**Mild**										
Tonic seizure	20		15		10	0	0	0	0	0	0	0	0	0	0
Hyperactivity or spasticity	15		10		5	0	0	0	0	0	0	0	0	0	0
Time required for:	**0.5 h**	**1 h**	**2 h**	**4 h**	**≥24 h**										
Maintenance of sternal posture	0	4	8	12	20	4	0	0	0	4	12	4	0	0	12
Unstimulated Movement	0	3	6	9	15	0	0	0	0	0	9	9	0	0	9
Movement without Ataxia	0	2	4	6	10	4	4	4	4	4	6	10	0	4	6
Normal Grooming/Eating/Nesting	0	1	2	3	5	3	3	3	3	3	5	5	3	3	5
Total Score						**11**	**7**	**7**	**7**	**11**	**32**	**28**	**3**	**7**	**32**

Table S14 continued (part 5/7).

ASO						aCSF (5Ca, 3Mg)				
Mouse ID						*8202*	*8205*	*8208*	*8211*	*8214*
Death			75			0	0	0	0	0
	Severe		Moderate		Mild					
Tonic seizure	20		15		10	0	0	0	0	0
Hyperactivity or spasticity	15		10		5	0	0	0	0	0
Time required for:	0.5 h	1 h	2 h	4 h	≥24 h					
Maintenance of sternal posture	0	4	8	12	20	0	0	0	0	0
Unstimulated Movement	0	3	6	9	15	0	0	0	0	0
Movement without Ataxia	0	2	4	6	10	4	2	2	2	0
Normal Grooming/Eating/Nesting	0	1	2	3	5	5	1	3	3	5
Total Score						**9**	**3**	**5**	**5**	**5**

ASO						aCSF (3.6Ca)									
Mouse ID						*8203*	*8206*	*8209*	*8212*	*8215*	*8916*	*8919*	*8922*	*8925*	*8928*
Death			75			0	0	0	0	0	0	0	0	0	0
	Severe		Moderate		Mild										
Tonic seizure	20		15		10	0	0	0	0	0	0	0	0	0	0
Hyperactivity or spasticity	15		10		5	0	0	0	0	0	0	0	0	0	0
Time required for:	0.5 h	1 h	2 h	4 h	≥24 h										
Maintenance of sternal posture	0	4	8	12	20	0	0	0	0	0	0	0	0	0	0
Unstimulated Movement	0	3	6	9	15	0	0	0	0	0	0	0	0	0	0
Movement without Ataxia	0	2	4	6	10	6	6	6	4	4	2	2	0	2	2
Normal Grooming/Eating/Nesting	0	1	2	3	5	5	5	5	3	5	2	2	2	2	3
Total Score						**11**	**11**	**11**	**7**	**9**	**4**	**4**	**2**	**4**	**5**

Table S14 continued (part 6/7).

ASO						aCSF (3.6Mg)									
Mouse ID						*8216*	*8217*	*8218*	*8219*	*8220*	*8957*	*8952*	*8953*	*8954*	*8955*
Death			75			0	0	0	0	0	0	0	0	0	0
	Severe		Moderate		Mild										
Tonic seizure	20		15		10	0	0	0	0	0	0	0	0	0	0
Hyperactivity or spasticity	15		10		5	0	0	0	0	0	0	0	0	0	0
Time required for:	0.5 h	1 h	2 h	4 h	≥24 h										
Maintenance of sternal posture	0	4	8	12	20	0	0	0	0	0	0	0	12	0	0
Unstimulated Movement	0	3	6	9	15	0	0	0	0	0	0	0	9	0	0
Movement without Ataxia	0	2	4	6	10	2	0	0	0	0	6	6	6	0	2
Normal Grooming/Eating/Nesting	0	1	2	3	5	0	3	3	2	2	5	5	5	5	5
Total Score						**2**	**3**	**3**	**2**	**2**	**11**	**11**	**32**	**5**	**7**

ASO						aCSF (empty)									
Mouse ID						*8248*	*8251*	*8254*	*8257*	*8260*	*8918*	*8921*	*8924*	*8927*	*8930*
Death			75			0	0	0	0	0	0	0	0	0	0
	Severe		Moderate		Mild										
Tonic seizure	20		15		10	0	0	0	0	0	0	0	0	0	0
Hyperactivity or spasticity	15		10		5	0	0	0	0	0	0	0	0	0	0
Time required for:	0.5 h	1 h	2 h	4 h	≥24 h										
Maintenance of sternal posture	0	4	8	12	20	0	0	0	8	8	0	0	0	0	0
Unstimulated Movement	0	3	6	9	15	0	0	0	6	6	0	0	0	0	0
Movement without Ataxia	0	2	4	6	10	2	4	0	4	4	0	0	0	0	2
Normal Grooming/Eating/Nesting	0	1	2	3	5	3	3	3	3	3	2	0	1	2	1
Total Score						**5**	**7**	**3**	**21**	**21**	**2**	**0**	**1**	**2**	**3**

Table S14 continued (part 7/7).

ASO						LRS				
**Mouse ID**						***8917***	***8920***	***8923***	***8926***	***8929***
**Death**			75			0	0	0	0	0
	**Severe**		**Moderate**		**Mild**					
**Tonic seizure**	20		15		10	0	0	0	0	0
**Hyperactivity or spasticity**	15		10		5	0	0	0	0	0
**Time required for:**	**0.5 h**	**1 h**	**2 h**	**4 h**	**≥24 h**					
**Maintenance of sternal posture**	0	4	8	12	20	0	0	0	0	0
**Unstimulated Movement**	0	3	6	9	15	0	0	0	0	0
**Movement without Ataxia**	0	2	4	6	10	0	2	0	0	4
**Normal Grooming/Eating/Nesting**	0	1	2	3	5	2	3	2	1	2
**Total Score**						**2**	**5**	**2**	**1**	**6**

ASO						LRS (empty)				
**Mouse ID**						***35***	***38***	***41***	***44***	***47***
**Death**			75			0	0	0	0	0
	**Severe**		**Moderate**		**Mild**					
**Tonic seizure**	20		15		10	0	0	0	0	0
**Hyperactivity or spasticity**	15		10		5	0	0	0	0	0
**Time required for:**	**0.5 h**	**1 h**	**2 h**	**4 h**	**≥24 h**					
**Maintenance of sternal posture**	0	4	8	12	20	0	0	0	0	0
**Unstimulated Movement**	0	3	6	9	15	0	3	0	0	0
**Movement without Ataxia**	0	2	4	6	10	0	2	0	0	0
**Normal Grooming/Eating/Nesting**	0	1	2	3	5	2	0	2	2	2
**Total Score**						**2**	**5**	**2**	**2**	**2**

Table S15. Raw data from the EvADINT scoring assay for each mouse in Figure S1a. (part 1/2)

ASO						*C9orf72* full PS w/Ca2+ (35nmol)					
**Mouse ID**						**47**	**48**	**49**	**50**	**51**	**52**
**Death**			75			0	0	0	0	0	0
	**Severe**		**Moderate**		**Mild**						
**Tonic seizure**	20		15		10	0	0	0	0	0	0
**Hyperactivity or spasticity**	15		10		5	10	0	5	5	0	0
**Time required for:**	**0.5 h**	**1 h**	**2 h**	**4 h**	**≥24 h**						
**Maintenance of sternal posture**	0	4	8	12	20	8	0	12	8	0	12
**Unstimulated Movement**	0	3	6	9	15	9	0	9	9	0	9
**Movement without Ataxia**	0	2	4	6	10	6	4	10	10	2	10
**Normal Grooming/Eating/Nesting**	0	1	2	3	5	3	3	5	5	0	5
**Total Score**						**36**	**7**	**41**	**37**	**2**	**36**

ASO						*C9orf72* full PS w/Ca2+ (15nmol)					
**Mouse ID**						**176**	**177**	**178**	**179**	**180**	**181**
**Death**			75			0	0	0	0	0	0
	**Severe**		**Moderate**		**Mild**						
**Tonic seizure**	20		15		10	0	0	0	0	0	0
**Hyperactivity or spasticity**	15		10		5	0	0	0	0	0	0
**Time required for:**	**0.5 h**	**1 h**	**2 h**	**4 h**	**≥24 h**						
**Maintenance of sternal posture**	0	4	8	12	20	0	0	0	0	0	0
**Unstimulated Movement**	0	3	6	9	15	0	0	0	0	0	0
**Movement without Ataxia**	0	2	4	6	10	6	6	6	6	6	6
**Normal Grooming/Eating/Nesting**	0	1	2	3	5	0	1	1	1	0	1
**Total Score**						**6**	**7**	**7**	**7**	**6**	**7**

ASO						*C9orf72* PO/PS w/Ca2+ (35nmol)					
**Mouse ID**						**25**	**26**	**27**	**28**	**29**	**30**
**Death**			75			0	0	0	0	0	0
	**Severe**		**Moderate**		**Mild**						
**Tonic seizure**	20		15		10	0	0	0	0	0	0
**Hyperactivity or spasticity**	15		10		5	0	0	0	0	0	0
**Time required for:**	**0.5 h**	**1 h**	**2 h**	**4 h**	**≥24 h**						
**Maintenance of sternal posture**	0	4	8	12	20	0	0	0	0	0	0
**Unstimulated Movement**	0	3	6	9	15	0	3	3	6	0	0
**Movement without Ataxia**	0	2	4	6	10	0	6	6	6	6	6
**Normal Grooming/Eating/Nesting**	0	1	2	3	5	2	1	2	2	1	1
**Total Score**						**2**	**10**	**11**	**14**	**7**	**7**

Table S15 continued (part 2/2).

ASO						C9orf72 PO/PS w/Ca2+ (15nmol)					
**Mouse ID**						**182**	**183**	**184**	**185**	**186**	**187**
**Death**	75					0	0	0	0	0	0
	**Severe**		**Moderate**		**Mild**						
**Tonic seizure**	20		15		10	0	0	0	0	0	0
**Hyperactivity or spasticity**	15		10		5	0	0	0	0	0	0
**Time required for:**	**0.5 h**	**1 h**	**2 h**	**4 h**	**≥24 h**						
**Maintenance of sternal posture**	0	4	8	12	20	0	0	0	0	0	0
**Unstimulated Movement**	0	3	6	9	15	0	0	0	0	0	0
**Movement without Ataxia**	0	2	4	6	10	6	6	6	6	6	6
**Normal Grooming/Eating/Nesting**	0	1	2	3	5	0	0	0	0	0	0
**Total Score**						**6**	**6**	**6**	**6**	**6**	**6**

ASO						PBS							
**Mouse ID**						**33**	**34**	**35**	**36**	**37**	**38**	**39**	**40**
**Death**	75					0	0	0	0	0	0	0	0
	**Severe**		**Moderate**		**Mild**								
**Tonic seizure**	20		15		10	0	0	0	0	0	0	0	0
**Hyperactivity or spasticity**	15		10		5	0	0	0	0	0	0	0	0
**Time required for:**	**0.5 h**	**1 h**	**2 h**	**4 h**	**≥24 h**								
**Maintenance of sternal posture**	0	4	8	12	20	0	0	0	0	0	0	0	0
**Unstimulated Movement**	0	3	6	9	15	0	0	0	0	0	0	0	0
**Movement without Ataxia**	0	2	4	6	10	0	0	0	0	0	0	0	2
**Normal Grooming/Eating/Nesting**	0	1	2	3	5	1	1	0	1	1	1	1	0
**Total Score**						**1**	**1**	**0**	**1**	**1**	**1**	**1**	**2**

Table S16. Raw data from the EvADINT scoring assay for each mouse in Figure S1b (part 1/2)

ASO						*Malat1* Full PS w/Ca2+ (35nmol)					
**Mouse ID**						**111**	**112**	**113**	**114**	**115**	**116**
**Death**			75			0	0	0	0	0	0
	**Severe**		**Moderate**		**Mild**						
**Tonic seizure**	20		15		10	10	10	10	10	10	10
**Hyperactivity or spasticity**	15		10		5	0	0	0	0	0	0
**Time required for:**	**0.5 h**	**1 h**	**2 h**	**4 h**	**≥24 h**						
**Maintenance of sternal posture**	0	4	8	12	20	12	20	0	20	20	20
**Unstimulated Movement**	0	3	6	9	15	9	15	9	15	15	15
**Movement without Ataxia**	0	2	4	6	10	10	10	10	10	10	10
**Normal Grooming/Eating/Nesting**	0	1	2	3	5	3	5	3	5	5	5
**Total Score**						**44**	**60**	**32**	**60**	**60**	**60**

ASO						*Malat1* Full PS w/Ca2+ (15nmol)					
**Mouse ID**						**188**	**189**	**190**	**191**	**192**	**193**
**Death**			75			0	0	0	0	0	0
	**Severe**		**Moderate**		**Mild**						
**Seizure**	20		15		10	0	10	10	0	10	10
**Hyperactivity or spasms**	15		10		5	10	0	0	10	0	0
**Time required for:**	**0.5 h**	**1 h**	**2 h**	**4 h**	**≥24 h**						
**Maintenance of sternal posture**	0	4	8	12	20	0	8	12	12	12	12
**Unstimulated Movement**	0	3	6	9	15	0	9	15	15	15	15
**Movement without Ataxia**	0	2	4	6	10	10	10	10	10	10	10
**Normal Grooming/Eating/Nesting**	0	1	2	3	5	2	5	5	5	5	5
**Total Score**						**22**	**42**	**52**	**52**	**52**	**52**

ASO						*Malat1* PO/PS w/Ca2+ (35nmol)					
**Mouse ID**						**123**	**124**	**125**	**126**	**127**	**128**
**Death**			75			0	0	0	0	0	0
	**Severe**		**Moderate**		**Mild**						
**Seizure**	20		15		10	0	0	0	0	0	0
**Hyperactivity or spasms**	15		10		5	5	0	0	0	0	0
**Time required for:**	**0.5 h**	**1 h**	**2 h**	**4 h**	**≥24 h**						
**Maintenance of sternal posture**	0	4	8	12	20	0	0	8	0	0	12
**Unstimulated Movement**	0	3	6	9	15	9	6	9	6	0	9
**Movement without Ataxia**	0	2	4	6	10	6	10	10	10	4	10
**Normal Grooming/Eating/Nesting**	0	1	2	3	5	3	2	3	3	1	3
**Total Score**						**23**	**18**	**30**	**19**	**5**	**34**

Table S16 continued (part 2/2).

ASO						*Malat1* PO/PS w/Ca2+ (15nmol)					
**Mouse ID**						**194**	**195**	**196**	**197**	**198**	**199**
**Death**			75			0	0	0	0	0	0
	**Severe**		**Moderate**		**Mild**						
**Seizure**	20		15		10	0	0	0	0	0	0
**Hyperactivity or spasms**	15		10		5	0	0	0	5	0	0
**Time required for:**	**0.5 h**	**1 h**	**2 h**	**4 h**	**≥24 h**						
**Maintenance of sternal posture**	0	4	8	12	20	0	0	0	0	0	0
**Unstimulated Movement**	0	3	6	9	15	0	0	0	3	0	0
**Movement without Ataxia**	0	2	4	6	10	6	6	6	6	6	6
**Normal Grooming/Eating/Nesting**	0	1	2	3	5	0	0	0	2	1	1
**Total Score**						**6**	**6**	**6**	**16**	**7**	**7**

ASO						PBS					
**Mouse ID**						**200**	**201**	**202**	**203**	**204**	**205**
**Death**			75			0	0	0	0	0	0
	**Severe**		**Moderate**		**Mild**						
**Seizure**	20		15		10	0	0	0	0	0	0
**Hyperactivity or spasms**	15		10		5	0	0	0	0	0	0
**Time required for:**	**0.5 h**	**1 h**	**2 h**	**4 h**	**≥24 h**						
**Maintenance of sternal posture**	0	4	8	12	20	0	0	0	0	0	0
**Unstimulated Movement**	0	3	6	9	15	0	0	0	0	0	0
**Movement without Ataxia**	0	2	4	6	10	2	2	2	4	2	2
**Normal Grooming/Eating/Nesting**	0	1	2	3	5	0	0	0	1	0	0
**Total Score**						**2**	**2**	**2**	**5**	**2**	**2**
